# Epidemiology of heart failure in diabetes: a disease in disguise

**DOI:** 10.1007/s00125-023-06068-2

**Published:** 2024-02-09

**Authors:** Anna G. Hoek, Elisa Dal Canto, Eva Wenker, Navin Bindraban, M. Louis Handoko, Petra J. M. Elders, Joline W. J. Beulens

**Affiliations:** 1 Epidemiology and Data Science, Amsterdam UMC, location Vrije Universiteit Amsterdam, Amsterdam, the Netherlands; 2 Amsterdam Cardiovascular Sciences, Amsterdam UMC, Amsterdam, the Netherlands; 3 Department of Experimental Cardiology, University Medical Center Utrecht, Utrecht, the Netherlands; 4 Julius Center for Health Sciences and Primary Care, University Medical Center Utrecht, Utrecht, the Netherlands; 5 Heartcenter, Department of Cardiology, Amsterdam UMC, location AMC, Amsterdam, the Netherlands; 6 Heartcenter, Department of Cardiology, Amsterdam UMC, location Vrije Universiteit Amsterdam, Amsterdam, the Netherlands; 7 Department of General Practice, Amsterdam UMC, location Vrije Universiteit Amsterdam, Amsterdam, the Netherlands; 8 Amsterdam Public Health, Amsterdam UMC, Amsterdam, the Netherlands

**Keywords:** Clinical research, Diabetes, Heart failure, diastolic, Heart failure, systolic, Lifestyle, Meta-analysis, Review, Systematic review

## Abstract

Left ventricular diastolic dysfunction (LVDD) without symptoms, and heart failure (HF) with preserved ejection fraction (HFpEF) represent the most common phenotypes of HF in individuals with type 2 diabetes mellitus, and are more common than HF with reduced ejection fraction (HFrEF), HF with mildly reduced ejection fraction (HFmrEF) and left ventricular systolic dysfunction (LVSD) in these individuals. However, diagnostic criteria for HF have changed over the years, resulting in heterogeneity in the prevalence/incidence rates reported in different studies. We aimed to give an overview of the diagnosis and epidemiology of HF in type 2 diabetes, using both a narrative and systematic review approach; we focus narratively on diagnosing (using the 2021 European Society of Cardiology [ESC] guidelines) and screening for HF in type 2 diabetes. We performed an updated (2016–October 2022) systematic review and meta-analysis of studies reporting the prevalence and incidence of HF subtypes in adults ≥18 years with type 2 diabetes, using echocardiographic data. Embase and MEDLINE databases were searched and data were assessed using random-effects meta-analyses, with findings presented as forest plots. From the 5015 studies found, 209 were screened using the full-text article. In total, 57 studies were included, together with 29 studies that were identified in a prior meta-analysis; these studies reported on the prevalence of LVSD (*n*=25 studies, 24,460 individuals), LVDD (*n*=65 studies, 25,729 individuals), HFrEF (*n*=4 studies, 4090 individuals), HFmrEF (*n*=2 studies, 2442 individuals) and/or HFpEF (*n*=8 studies, 5292 individuals), and on HF incidence (*n*=7 studies, 17,935 individuals). Using Hoy et al’s risk-of-bias tool, we found that the studies included generally had a high risk of bias. They showed a prevalence of 43% (95% CI 37%, 50%) for LVDD, 17% (95% CI 7%, 35%) for HFpEF, 6% (95% CI 3%, 10%) for LVSD, 7% (95% CI 3%, 15%) for HFrEF, and 12% (95% CI 7%, 22%) for HFmrEF. For LVDD, grade I was found to be most prevalent. Additionally, we reported a higher incidence rate of HFpEF (7% [95% CI 4%, 11%]) than HFrEF 4% [95% CI 3%, 7%]). The evidence is limited by the heterogeneity of the diagnostic criteria over the years. The systematic section of this review provides new insights on the prevalence/incidence of HF in type 2 diabetes, unveiling a large pre-clinical target group with LVDD/HFpEF in which disease progression could be halted by early recognition and treatment.

*Registration* PROSPERO ID CRD42022368035.

**Graphical Abstract:**

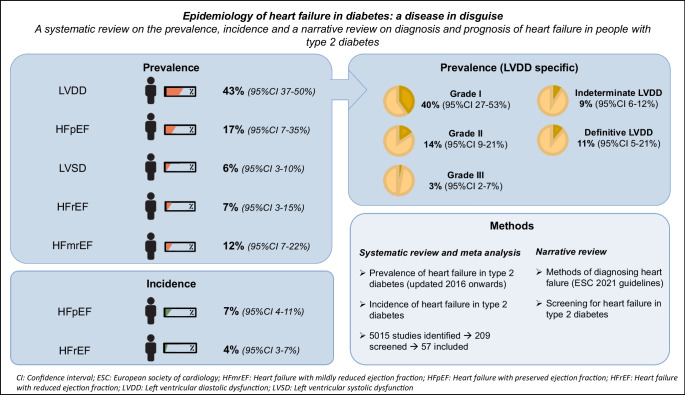

**Supplementary Information:**

The online version contains peer-reviewed but unedited supplementary material including a slideset of the figures for download, which is available to authorised users at 10.1007/s00125-023-06068-2.

## Introduction

Heart failure (HF) and type 2 diabetes are two highly intertwined diseases that exist in a vicious circle; people with type 2 diabetes are approximately two times more likely to develop HF than those without [1–4]. Furthermore, 30–40% of people with HF suffer from type 2 diabetes or show signs of impaired glucose tolerance, with the rate increasing to up to 50% in patients hospitalised for HF [5, 6]. Given an estimated prevalence of 537 million cases for diabetes [7] and 64.3 million cases for HF [8] worldwide, the risks of hospitalisation, CVD-attributable mortality and all-cause mortality in people with both of these diseases represent an increasing burden on healthcare, including healthcare-related costs [9, 10].

Rather than being an encapsulated disease, HF should be viewed as a heterogeneous syndrome, consisting of multiple clinical entities and different stages. HF can be categorised as HF with reduced ejection fraction (HFrEF; left ventricle ejection fraction [LVEF], ≤40%), HF with mildly reduced ejection fraction (HFmrEF; LVEF, 41–49%) and HF with preserved ejection fraction (HFpEF; LVEF, ≥50%). Furthermore, echocardiographically distinct phenotypes of ventricular dysfunction in systole (left ventricular systolic dysfunction [LVSD]) and in diastole (left ventricular diastolic dysfunction [LVDD]) can be identified; these reflect ventricular dysfunction without clinical symptomatology of HF [11]. Out of these categories, LVDD and HFpEF currently represent the most common phenotypes of HF in type 2 diabetes, although there is no consensus on the exact prevalence of the HF subtypes [12, 13].

Since pathophysiology, treatment and prognosis differ depending on the subtype of HF [14], a timely and accurate diagnosis of HF (subtype) and identification of people at risk for HF is important. This is even more true for people with type 2 diabetes, since sodium−glucose cotransporter 2 (SGLT2) inhibitors provide both glucose lowering and cardiovascular protection, showing promising effects on cardiovascular outcomes [15, 16]. Knowledge about the exact prevalence and incidence of HF and its pre-clinical stages is a key factor in the process of accurately diagnosing HF.

In this review, we provide an overview of the epidemiology and diagnostic process of HF in individuals with type 2 diabetes, covering diagnosis, screening and prognosis in a narrative way. Furthermore, we report the findings from an updated systematic review and meta-analysis of the study by Bouthoorn et al [12, 13] on the prevalence of LVSD, LVDD, HFpEF, HFrEF and HFmrEF, including studies published from 2016 onwards. We also present results from a novel systematic review and meta-analysis on the incidence of HF subtypes in type 2 diabetes. In doing so, we aim to provide the most updated numbers on prevalence and incidence of HF in type 2 diabetes.

## Diagnosis of HF in type 2 diabetes

Over the years, many algorithms and guidelines have been proposed to ease the process of clinically diagnosing and categorising HF. Nevertheless, much controversy remains, especially about the diagnosis of LVDD/HFpEF. In 2021, the European Society of Cardiology (ESC) published guidelines for the diagnosis and treatment of HF [17]. In this section, we aim to give an overview of the diagnostic process based on these guidelines.

**Fig. 1:**
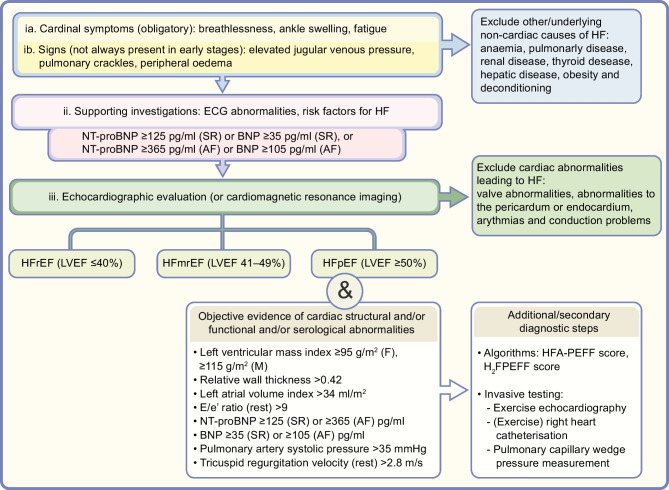
Schematic overview of diagnostic pathway for HF in people with type 2 diabetes, based on the 2021 ESC guidelines for the diagnosis and treatment of HF. HF can be categorised as HFrEF, HFmrEF or HFpEF. The diagnosis of HF requires the presence of cardinal symptoms (ia) and, occasionally, signs (ib). Supporting investigations (ii) include ECG, risk factor assessment (risk factors include medical history of cardiovascular events, older age [>70 years], sex and obesity) and analysis of NP levels (note that in individuals with atrial fibrillation [AF], diagnostic values are ≥365 pg/ml N-terminal pro–B-type NP [NT-proBNP] and ≥105 pg/ml brain NP [BNP]). Echocardiography (iii) allows for differentiation into different categories of HF. A reduced LVEF is needed to diagnose HFrEF and HFmrEF (≤40% for HFrEF and 41–49% for HFmrEF), and a preserved LVEF (≥50%) combined with echocardiographic functional and/or structural abnormalities and/or serological abnormalities is required for the diagnosis of HFpEF. Secondary diagnostic tests may include use of diagnostic algorithms, which are non-invasive. More invasive tests (e.g., exercise echocardiography, [exercise] right heart catheterisation and/or pulmonary artery wedge pressure) may be used if HF is suspected despite normal results for tests of echocardiographic functional and/or structural abnormalities and serological abnormalities, and if other comorbidities do not sufficiently explain symptoms/signs. F, female; M, male; SR, sinus rhythm. This figure is available as part of a downloadable slideset

For diagnosing HF, the presence of cardinal symptoms (e.g., breathlessness, ankle swelling and/or fatigue) are obligatory and might, in more advanced clinical stages, be accompanied with signs of HF (e.g., elevated jugular venous pressure, pulmonary crackles, peripheral oedema) (Fig. 1). Questionnaires, such as the Kansas City Cardiomyopathy questionnaire [18] and the Minnesota Living with Heart Failure Questionnaire [19], can be used to assess symptoms in a validated manner. Furthermore, in this stage, non-cardiac diseases (that can coexist with HF and exacerbate the HF syndrome) such as anaemia, and pulmonary, renal, thyroid or hepatic disease should be excluded.

Additionally, the determination of risk factors for HF (e.g. a medical history of cardiovascular events, older age [>70 years], sex and obesity) and an abnormal ECG can support clinical suspicion of HF. Natriuretic peptides (NPs) play a key role as initial diagnostic markers, and the ESC guidelines state that elevated NP concentrations (N-terminal pro–B-type NP [NT-proBNP] ≥125 pg/ml [≥365 pg/ml in individuals with atrial fibrillation]; brain NP [BNP] ≥35 pg/ml [≥105 pg/ml in atrial fibrillation]) support a diagnosis of HF (Fig. 1) [17]. For HFrEF and LVSD, the sensitivity and negative predictive value of ECG and NP analysis to detect cardiac disease are high [17, 20, 21], but they appear less reliable for diagnosing HFpEF [22–24]. A meta-analysis reported low sensitivity and specificity for the detection of LVDD and HFpEF based on ECG and NP analysis (sensitivity: 65% [95% CI 51%, 85%]; specificity: 80% [95% CI 70%, 90%]), accompanied by a reasonable ability to rule out LVDD (negative predictive value: 85% [95% CI 78%, 93%]) but poor positive-predictive value (60% [95% CI 30%, 90%]) [23]. Furthermore, NPs tend to be increased in the older population, relate inversely to BMI, are affected by kidney function and can be falsely elevated. Therefore, even though NP levels can be good indicators for HF, diagnosis of HF cannot be made or omitted based on NP measurements alone.

Echocardiography is key in the initial diagnostic work-up as it provides information about LVEF and the underlying aetiology (e.g., ischaemic, valvular) [17]. The diagnosis of HFrEF and HFmrEF requires the presence of symptoms (and, optionally, signs) of HF, as well as a reduced LVEF (≤40% for HFrEF and 41–49% for HFmrEF). However, the diagnosis of HFpEF remains challenging. Before 2021, several diagnostic guidelines/algorithms had been proposed to diagnose HFpEF, of which the H_2_FPEF algorithm and the Heart Failure Association Pre-test assessment, Echocardiography and natriuretic peptide, Functional testing, Final etiology (HFA-PEFF) score, together with the 2016 American Society of Echocardiography (ASE)/European Association of Cardiovascular Imaging (EACVI) recommendation guidelines [25], are the most well-known [26, 27]. The ASE/EACVI guidelines use echocardiographic factors only, whilst the H_2_FPEF and HFA-PEFF algorithms use a combination of echocardiographic factors and, clinical factors/patient characteristics, and differentiate between low, intermediate or high probability of having HFpEF. However, the use of these algorithms is subject to interpretation and is reported in a heterogeneous way [28]. Furthermore, when the H_2_FPEF and HFA-PEFF algorithms were applied to the same population, a significant fraction of individuals were classified discordantly, with 41% of participants being placed in different likelihood categories by each of the two scores [29–31]. Validation studies show that the H_2_FPEF score has a superior diagnostic performance compared with the HFA-PEFF score [32]; nevertheless, neither are perfect discriminators.

In 2021, the ESC published guidelines that recommend a simplified pragmatic approach for HF diagnosis, using the common major elements from earlier algorithms but in a more accessible and clinician-friendly way [17]. This approach became the preferred diagnostic tool to use. It is based on clinical symptoms (and, optionally, signs), and the presence of either structural and/or functional abnormalities in people with a preserved ejection fraction (≥50%), which are assessed using echocardiographic variables that represent signs of LVDD and are relatively easily to access (Fig. 1). By use of this algorithm, HFpEF can be diagnosed in a relatively non-invasive way. Nevertheless, if HF is suspected despite normal results, and other comorbidities do not sufficiently explain symptoms/signs, diastolic stress tests [33], such as exercise echocardiography [34] and/or (exercise) right heart catheterisation/assessment of pulmonary capillary wedge pressure [33, 35, 36], are recommended.

## The prevalence and incidence of HF in type 2 diabetes: an updated systematic review

Knowledge on the prevalence of HF in people with type 2 diabetes is essential to identify a population at high risk. Nevertheless, a consensus has not been reached on the precise prevalence of (undiagnosed) HF and its subtypes in the type 2 diabetes population. In 2016–2018, Bouthoorn et al performed two meta-analyses on the prevalence of HF and left ventricular dysfunction [12, 13]; these analyses included a total of 29 studies. For LVDD, they found a prevalence of 35% (95% CI 24%, 46%) in the general population and 48% (95% CI 38%, 59%) in the hospital population [37–60], whilst for HFpEF, they reported a prevalence of 25% (95% CI 21%, 28%) in the general population [48] and 8% (95% CI 5%, 14%) in the hospital population [61]. For LVSD, they reported a prevalence of 2% (95% CI 2%, 3%) in the general population and 18% (95% CI 17%, 19%) in the hospital population [38–42, 44, 47–49, 52, 56, 57, 59, 62–65] and, finally, for HFrEF, they found a prevalence of 5.8% (95% CI 3.9%, 7.6%) in the general population (based on one study) [48]. Since the publication of the meta-analyses by Bouthoorn and colleagues, new diagnostic guidelines have become available, allowing for more precise prevalence estimates. Therefore, we have updated the search from Bouthoorn et al including studies from 2016 to 20 October 2022. We used the same search strategy as Bouthoorn et al with terms for HF (e.g., HFpEF, HFrEF, systolic, diastolic), echocardiography and diabetes/type 2 diabetes (see electronic supplementary material [ESM] Methods, ‘Search strategies’ section), and we included studies reporting on prevalence and incidence of cardiac dysfunction/HF based on echocardiographic measurements. Additionally, we meta-analysed the prevalence of LVDD categorically (grade I, II, III and/or indeterminate/definitive LVDD) when this information was available, and we performed a sensitivity analysis on the prevalence of LVDD, which only included studies that used a cut-off of LVEF ≥50%, to adhere to the most recent guidelines. Methodological quality assessment of the included studies was performed; this was based on Hoy et al’s risk-of-bias tool [66]. A detailed description of the methods used can be found in the ‘Systematic review and meta-analysis’ section of the ESM Methods. Initial screening was done by three authors (AGH, JWJB and EW) and selection was done by two authors (AGH, and JWJB). Data extraction/risk-of-bias assessment was done by AGH and was performed in twofold for 25% of the extracted papers (JWJB and EDC), with an excellent agreement for data extraction (absolute agreement: 98%) and a good agreement for risk of bias (absolute agreement on final score: 74%; note that Hoy et al reported an agreement value of 72% in the validation process in their study [66]). Screening and selection was done independently and consensus was used to resolve disagreement. There were no automation tools used. The Preferred Reporting Items for Systematic Reviews and Meta-Analyses (PRISMA) guidelines were followed [67], and the completed checklists can be found in ESM Tables 1 and 2. The protocol for this review was registered in the International Prospective Register of Systematic Reviews, the PROSPERO database, under number: CRD42022368035.

**Fig. 2:**
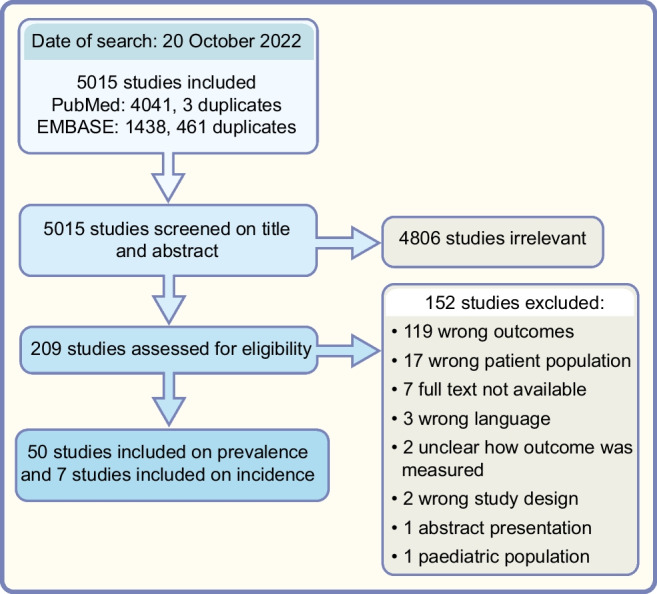
PRISMA flow chart showing the process for selection of relevant articles included in the systematic review and meta-analysis. This figure is available as part of a downloadable slideset

Of the 5015 unique studies identified, 209 were screened using the full-text article and, in total, 50 studies reported on prevalence of LVSD (*n*=8) [68–75], LVDD (*n*=41) [68, 70, 71, 74, 76–112], HFrEF (*n*=3) [113–115], HFmrEF (*n*=2) [113, 114] and HFpEF (*n*=6) [73, 106, 113, 115–117] were included in this updated review and meta-analysis (Table 1). A PRISMA flow diagram of the process for selection of relevant articles is presented in Fig. 2. Eight studies included participants derived from the general population or a primary-care population [68, 69, 89, 92, 93, 102, 103, 111], three studies did not specify where the participants with type 2 diabetes were selected from [94, 104, 105] and the remaining studies all included patients with type 2 diabetes from a hospital setting (cardiology/endocrinology departments) or specialised outpatients clinics that focused on either cardiology/endocrinology [70–88, 90, 91, 95–102, 104–110, 113–115]. All but three [94, 98, 104] studies reported the mean age of their participants, ranging from 44.5 years to 76.2 years (Table 1). Diabetes duration was reported in 30 studies and ranged from a mean of 3 years to 14.8 years (or 17.9 years in a subgroup of the study by Zoppini et al [87]) (Table 1). Due to a lack of consensus on how to diagnose LVDD and HFpEF in the past, the method used for diagnosing these conditions varied largely between studies; an overview of the criteria used to diagnose LVDD and HFpEF in each study is given in ESM Table 3 and ESM Table 4, respectively.

**Table 1:** Characteristics and quality assessment of the included studies

Authors (year of publication)	General study characteristics					Risk of bias^b^									
	Source population and setting	Age of population, years^a^	Participants with T2D, *n* (% male)	T2D duration, years^a^	Exclusion criteria (shortened)	(a)	(b)	(c)	(d)	(e)	(f)	(g)	(h)	(i)	Overall risk
Alhibaly et al (2021) [76]	Outpatient clinic (cardiac clinic, patients with an indication for TTE)	50.5±15.0 for total population; not specified for T2D subgroup	65 (% male unknown)	Not reported	Not reported	Low	High	High	High	High	Low	Low	High	Low	High
Alizadehasl et al (2021) [68]	Population-based cohort	49.0±11.3 for total population; not specified for T2D subgroup	203 (% male unknown)	Not reported	History of CVD, including AMI	Low	Low	Low	High	High	Low	Low	High	Low	Medium
Antakly-Hanon et al (2021) [70]	Hospital population (endocrinology/diabetology)	57.5±11.0	200 (54.0%)	11.0 (6.0–19.0)	History of CVD, including AMI, active malignancy, radiation therapy	Low	Low	High	High	Low	Low	Low	High	Low	Medium
Bayat et al (2020) [77]	Hospital population (hospital department unspecified)	54.3±8.2	62 (59.7%)	Not reported	History of CVD, including AMI, EF <50%, uncontrolled BP, chronic renal, liver or lung disease, pregnancy	Low	High	High	High	High	Low	Low	High	High	High
Bergerot et al (2018) [95]	Hospital population (endocrinology/diabetology)	57.3±7.9	310 (52%)	12.0±7.8	History of CVD, including AMI, EF <55%, uncontrolled BP, chronic renal disease	Low	Low	Low	High	Low	Low	Low	Low	Low	Low
Chee et al (2021) [71]	Outpatient clinic (endocrinology/diabetology)	Mean (minimum, maximum): 61 (26, 86)	301 (34.6%)	<5: 6.98%; 5–10: 9.97%; >10: 83.05%	History of CVD, including AMI, HF, chronic renal disease, malignancy, inflammatory disease	Low	Low	Low	High	Low	Low	Low	High	Low	Medium
Cioffi et al (2021) [78]	Hospital population (participants were selected on having concentric LV geometry)	69±9	188 (55.8%)	7 (4–12)	This information (published as supplementary materials) could not be accessed	High	High	Low	High	?	?	Low	Low	Low	?
Demmer et al (2016) [102]	Population-based cohort	Mean±SEM: 58.9±0.5	511 (44.75%)	Not reported	Not reported	Low	Low	Low	High	Low	Low	Low	Low	Low	Low
Fudim et al (2019) [72]	Hospital population (hospital department unspecified)	64.0 (58.0–69.0) for individuals with HF; 62.0 (55.0–68.0) for individuals without HF	14,751 (62.02%)	12.0 (7.0–18.0)	Type 1 diabetes, prone to hypoglycaemia, renal disease	Low	Low	Low	Low	Low	Low	Low	High	High	Medium
Gimeno-Orna et al (2021) [113]	Hospital population (endocrinology/diabetology and cardiology)	67.3±10.1	1497 (67%)	14.0±11.1	Type 1 diabetes, chronic renal disease, life expectancy <3 years	Low	Low	Low	High	Low	Low	Low	Low	Low	Low
Huang et al (2022) [79]	Hospital population (patients with an indication for TTE)	58.97±7.25	1135 (56.74%)	Range: 4.54±5.22 to 7.15±7.36 in individuals stratified into quartiles based on baseline cystatin C levels	History of CVD, including AMI, HF, LVEF <50%, chronic renal disease, AF	Low	High	High	High	Low	Low	Low	High	Low	High
Ianos et al (2021) [116]	Hospital population (hospital department unspecified)	64.65±9.83	69 (53.62%)	Not reported	History of CVD, including AMI, HF, EF <50%, chronic renal or lung disease, malignancy, anaemia	Low	Low	High	High	Low	Low	Low	High	Low	Medium
Ibrahim et al (2021) [80]	Hospital population (cardiology)	49.07±6.10	90 (52.2%)	3 (1–5)	History of CVD, including AMI, HF, history of high BP, chronic renal disease, infectious disease, malignancy, pregnancy	Low	Low	High	High	Low	Low	Low	High	Low	Medium
Jensen et al (2019) [73]	Outpatients clinic (endocrinology/diabetology)	Normal TTE: 63.8 (55.6–69.2); abnormal TTE: 65.8 (59.6–71.8); HFpEF: 68.2 (62.7–74.3); HFrEF: 72.2 (64.9–76.6)	806 (65.14%)	Normal TTE: 10.0 (5.0–15.0); abnormal TTE: 12.0 (6.9–18.2); HFpEF: 15.0 (8.0–22.0); HFrEF: 12.0 (10.0–23.0)	AF, heart valve replacement	Low	Low	Low	High	Low	Low	Low	High	Low	Medium
Joseph et al (2020) [96]	Outpatients clinic (endocrinology/diabetology)	44.5 (40.0–47.0)	62 (71%)	8.5 (6.0–11.0)	History of CVD, including AMI, HF, chronic renal disease, connective tissue disorder, pregnancy	Low	Low	High	High	High	Low	Low	High	Low	High
Kasha et al (2017) [104]	Not specified	<50 years	50 (% male unknown)	Not reported	>50 years old, history of CVD, renal failure, hypertension	High	High	High	High	High	Low	Low	High	High	High
Kim et al (2019) [89]	Population-based cohort	55.4±7.3 for normal-weight individuals; 54.5±6.2 for individuals with obesity	219 (61.2%)	Not reported	Underweight individuals, use of antihypertensive medication, history of CVD and/or HF, kidney insufficiency	Low	Low	Low	High	Low	Low	Low	High	Low	Medium
Kishi et al (2017) [69]	Population-based cohort	50.6±3.7 for individuals with late diabetes (developed 15 years after inclusion in study); 51.2±3.1 for individuals with early diabetes (developed in year 0–14 after inclusion in study)	453 (45.7%)	>10 vs <10	Pregnancy, missing data	Low	Low	Low	High	High	Low	Low	High	Low	Medium
Klajda et al (2020) [103]	Population-based cohort	66.6±9.4	116 (58%)	Not reported	Not reported	Low	Low	Low	High	Low	Low	High	High	Low	Medium
Lee et al (2020) [90]	Hospital population (patients with an indication for TTE)	63.1±7.0	606 (23.6%)	8.4±7.0	History of CVD, alcohol consumption of >14 g/week, liver cirrhosis	High	High	High	High	Low	Low	Low	High	Low	High
Lee et al (2020) [91]	Outpatient clinic (patients with an indication for TTE)	62.0±15.1	100 (50%)	11.5±8.6	Type 1 diabetes, liver cirrhosis, renal disease, malignancy or any other serious illness	Low	High	High	High	High	Low	Low	High	Low	High
Li et al (2016) [117]	Hospital population (endocrinology/diabetology)	69.3±12.1	807 (57.4%)	10.2±8.3	History of CVD, EF <50%, chronic renal disease, disruptions in calcium and/or vitamin D metabolism, pulmonary disease, anaemia	Low	Low	High	High	Low	Low	Low	High	Low	Medium
Liu et al (2021) [81]	Hospital population, outpatients and inpatients (patients with an indication for TTE)	56.83±8.93 for individuals without LVDD; 58.62±7.15 for individuals with LVDD	327 (60.2%)	6 (4–9) for individuals without LVDD; 7 (5–10) for individuals with LVDD	History of CVD, including AMI, HF, history of high BP (or medication use for high BP), cancer	Low	High	High	High	Low	Low	Low	High	Low	High
Lu et al (2017) [82]	Hospital population (endocrinology/diabetology)	Mean±SEM: 76.2±0.9	154 (50%)	Not reported	>90 years old, BMI >40 kg/m^2^, known CVD, post pancreas transplantation	Low	High	High	High	High	Low	Low	High	Low	High
Lumori et al (2022) [97]	Hospital population (endocrinology/diabetology)	62.00±11.50	195 (27.69%)	10 (7–15)	HF, valvular disease, thyroid disorder, alcohol abuse	Low	Low	Low	High	Low	Low	Low	Low	Low	Low
Maiello et al (2017) [83]	Hospital population (endocrinology/diabetology)	66±9	665 (33.4%)	Not reported	History of CVD, including ACS, HF, type 1 diabetes, hypertension	Low	Low	Low	High	High	Low	Low	High	Low	Medium
McGuire et al (2019) [114]	Hospital population (patients with albuminuria)	65.90±9.10 for the total population	6979 (62.9%), of which 945 underwent echocardiographic evaluation	14.8±9.5 in the total population	Dialysis or eGFR <15 ml/min per 1.73m^2^, GLP-1 agonist/DPP-4 inhibitor or SGLT2 inhibitor use	High	High	Low	Low	Low	Low	Low	Low	Low	Medium
Nasir et al (2016) [98]	Hospital population (cardiology)	30–70 years (35.1% of individuals aged 50–59)	97 (50.5%)	0–9: 74.20%; 10–20: 23.70%; >20: 2.06%	IHD and/or hypertensive heart disease	Low	High	High	High	Low	Low	Low	High	Low	High
Oo et al (2021) [106]	Hospital population (department not specified)	62±10	305 (35.4%)	<5: 6.9%; 5–10: 9.8%; >10: 83.3%	History of CVD, deranged liver function, end stage renal failure	Low	High	High	High	Low	Low	Low	High	Low	Medium
Patro et al (2021) [74]	Outpatient clinic (diabetology/endocrinology patients with microalbuminuria)	53.70±5.32	62 (62.9%)	14.5±2.92	Severe hypertension, IHD, UTI, valvular heart disease, CKD and proteinuria >300 mg/24 h	Low	High	High	High	Low	Low	High	Low	Low	High
Qureshi et al (2016) [84]	Hospital population (endocrinology/diabetology, patients with NAFLD)	47.65±3.61	76 (57.9%)	Not reported	Myocardial infarction, valvular or rheumatic heart disease, chronic liver or renal disease, insulin use	Low	Low	High	High	Low	Low	Low	High	Low	Medium
Raghothama and Rao (2021) [107]	Outpatient and hospital population (endocrinology/diabetology)	56.52±9.94	217 (66.67%)	8.04±7.75 for individuals with LVDD; 5.27±5.49 for individuals without LVDD	History of CVD or symptoms of CVD (dyspnoea, angina, hypertension), type 1 diabetes. anaemia, history of smoking, history of significant alcohol intake, pregnancy, thyroid disease	Low	Low	High	High	Low	Low	Low	High	Low	Medium
Segar et al (2021) [92]	Population-based cohort	71.5±8.6	2900 (44.7%)	Not reported	History of CVD or HF (LVEF <45%), <40 years old	Low	Low	High	High	Low	Low	High	High	Low	High
Shahapure and Sharma (2020) [85]	Hospital population (endocrinology/diabetology patients with NAFLD)	52.81±4.96	100 (male/female ratio:1.17)	Not reported	History of CVD, hepatic cirrhosis, pregnancy, smokers, use of anti-cancer drugs, immunosuppressants and steroids	Low	Low	Low	High	High	Low	Low	High	Low	High
Shaker (2019) [94]	Not specified	Range: 20–64	80 (55%)	Not reported	History of cardiac disease, HF/systolic dysfunction, hypertension	High	High	High	High	Low	Low	Low	High	Low	High
Shogade et al (2018) [99]	Hospital population (endocrinology/diabetology and cardiology)	50±8	134 (47.8%)	4.7±2.8 for individuals with normoalbuminuria; 6.1±4.1 for individuals with microalbinuria	History of cardiac disease, hypertension, serum creatinine ≥114.38 µmol/l, chest deformity, sickle cell disease, UTI, pregnancy, >65 years old	Low	Low	Low	High	Low	Low	Low	High	Low	Low
Sunil Kumar et al (2021) [108]	Hospital and outpatient population	53±13 in the total population (including individuals without diabetes)	133 (63.4% male in total population, including individuals without T2D)	Not reported	History of CVD, including systolic dysfunction (LVEF <50%)	Low	Low	Low	High	Low	Low	Low	Low	Low	Low
Tremamunno et al (2022) [100]	Hospital population (endocrinology/diabetology)	64.1±10.0	84 (64.3%)	13.6±8.0	History of CVD, insulin use, liver and/or kidney failure, malignancies, chronic inflammatory disorders, neuromuscular and psychiatric diseases	Low	Low	Low	High	Low	Low	Low	High	Low	Low
Wan et al (2019) [111]	Population-based cohort	54.0±12.4 for individuals with normal diastolic function; 56.8±17.8 for individuals with diastolic dysfunction	307 (40.7%)	10.7±8.6 in individuals with normal diastolic function; 12.8±10.6 in individuals with diastolic dysfunction	Not reported	Low	Low	Low	High	Low	Low	Low	High	Low	Medium
Wang et al (2022) [86]	Hospital population (department not specified)	53.5±12.1 for individuals with normal diastolic function; 62.2±6.1 for individuals with indeterminate diastolic function; 65.5±6.3 for individuals with LVDD	90 (51.1%)	8.1±7.0 for individuals with normal diastolic function; 11.4±6.8 for individuals with indeterminate diastolic function; 12.2±6.2 for individuals with LVDD	History of cardiac disease, hypertension, anaemia, chronic obstructive pulmonary disease	Low	Low	High	High	Low	Low	Low	High	Low	Medium
Wang et al (2018) [93]	Population-based cohort	70.9±4.3	290 (56.2%)	Not reported	Existing HF or known IHD/reduced EF (<40%), more than moderate valve disease	Low	Low	Low	Low	Low	Low	Low	Low	Low	Low
Wang et al (2022) [112]	Hospital population (department not specified)	67.5±10.2	7112 (50.6%)	8 (6–9)	History of cardiac disease, thyroid disease, type 1 diabetes	Low	Low	High	High	Low	Low	High	High	Low	Medium
Wu et al (2018) [75]	Outpatient population, internal medicine (patients with stage 3–5 CKD)	66.2±11.8	208 (60.6%)	Not reported	Dialysis or significant mitral valve disease	Low	Low	Low	High	Low	Low	Low	High	Low	Medium
Wu et al (2021) [110]	Hospital population (department not specified)	61±11	350 (51.7%)	14±8	History of cardiac disease, including HF, malignancy, renal failure	Low	Low	Low	High	Low	Low	Low	High	Low	Medium
Yang et al (2016) [105]	Not specified	71.0±4.5	296 (55.4%)	Not reported	Cardiac disease, including HF with EF <40%, inability to get good quality echocardiographic images	Low	Low	Low	High	Low	Low	Low	High	Low	Low
Yang and Hwang (2022) [109]	Outpatient clinic (cardiac clinic, patients with an indication for TTE)	59±10	268 (65%)	Not reported for overall population	History of PCI, carotid artery bypass surgery, CVD (including systolic dysfunction [LVEF <40%]), type 1 diabetes, systemic disease (including kidney and liver disease)	Low	Low	Low	High	Low	Low	Low	Low	Low	Low
Zhen et al (2016) [101]	Hospital population (endocrinology/diabetology)	59.2±9.7	108 (52.8%)	13.6±6.3	History of CVD and/or abnormal treadmill stress test	Low	Low	Low	High	Low	Low	Low	Low	Low	Low
Zhou et al (2022) [115]	Hospital population (endocrinology/diabetology and cardiology; patients with an indication for TTE)	63.7±12.4	1043 (56.3%)	Not reported	Cardiac disease, HF	Low	Low	Low	Low	High	Low	Low	Low	Low	Low
Zoppini et al (2018) [87]	Endocrinology outpatient clinic	Group 0: 64.1±8.8; Group 1: 67.3±7.0; Group 2: 68.5±8.0; Group 3: 70.0±6.4^c^	176 (100%)	Group 0^c^: 8.4±9.4; Group 1^c^: 10.5±8.2; Group 2^c^: 17.9±11.2; Group 3^c^ 15.2±9.2	History of CVD, including HF (LVEF <50%), cirrhosis, malignancy or overt nephropathy	High	High	High	High	High	Low	Low	High	Low	Medium
Zuo et al (2019) [88]	Hospital population (endocrinology/diabetology)	57.09±11.86 for normal-weight individuals; 57.61±11.67 for individuals with overweight/obesity	925 (70.49%)	10.81±7.13 for normal-weight individuals; 9.85±7.09 for individuals with overweight/obesity	CVD, including LVEF <50%, individuals with active illness or who are underweight, pregnancy, renal insufficiency, eGFR <60 ml/min per 1.73m^2^	Low	Low	Low	High	High	Low	Low	High	Low	Medium

^a^Data presented as mean±SD or median (IQR), unless stated otherwise

^b^Assessed using Hoy et al’s risk-of-bias tool [66] with the following question: (a) do the included participants and settings match what is intended by the review question (i.e. individuals with type 2 diabetes from the general population, referral centres and hospital centres)? (b) Is the sampling frame a true or close representation of the population intended to be assessed by the review question? (c) Were an unselected (random/consecutive) sample of individuals invited to participate? (d) Was the response rate ≥75% or did a non-response analysis show no difference in response rate between participants and non-participants? (e) Was an acceptable case definition for LVDD and/or HFpEF used in the study? (f) Is the instrument used to measure LVDD and/or HFpEF valid? (g) Was the same mode of data collection used for all participants? (h) Is it unlikely that the handling of missing (endpoint) data introduced bias? (i) Were the numerator(s) and denominator(s) for the variables of interest appropriate? All signalling questions were scored as either low or high risk of bias. Studies were classified as having a low overall risk of bias if ≤1 question had a high risk of bias, a medium overall risk of bias if 2–3 questions had a high risk of bias, or high overall risk of bias if >3 questions had a high risk of bias

^c^Categorisation of participants according to median values of left ventricular end-diastolic volume (LVEDV)/body surface area (BSA) and E/e′ ratio: Group 0, LVEDV/ BSA <56 ml/m^2^ and average E/e′ ≤8; Group 1, LVEDV/BSA ≥56 ml/m^2^ and average E/e′ ≤8; group 2, LVEDV/BSA ≥56 ml/m^2^ and average E/e′ >8; group 3, LVEDV/BSA <56 ml/m^2^ and average E/e′ >8

ACS, acute coronary syndrome; AMI, acute myocardial infarction; CKD, chronic kidney disease; DPP-4, dipeptidyl peptidase-4; EF, ejection fraction; GLP-1, glucagon-like peptide 1; IHD, ischaemic heart disease; LV, left ventricular; NAFLD, non-alcoholic fatty liver disease; PCI, percutaneous coronary intervention; T2D, type 2 diabetes; TTE, transthoracic echocardiogram; UTI, urinary tract infection

### LVSD, HFmrEF and HFrEF

**Fig. 3:**
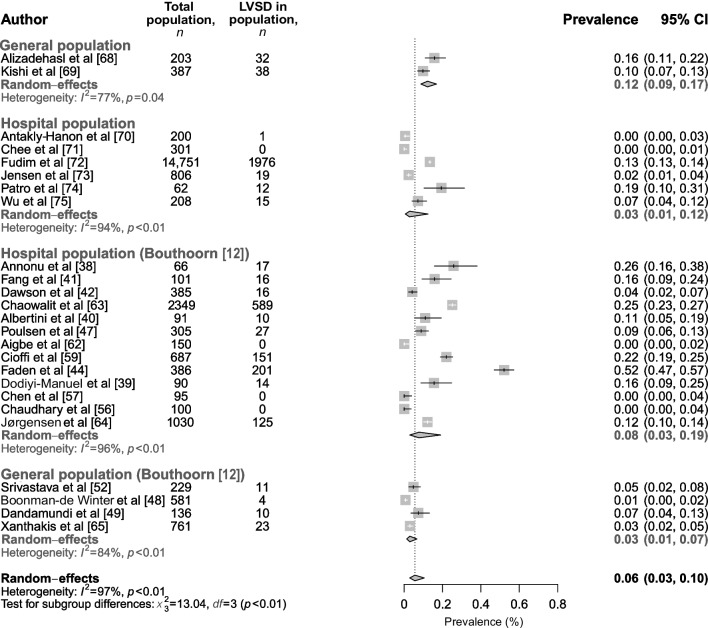
Prevalence of LVSD in individuals with type 2 diabetes in the hospital and general population, categorised by new studies included in this updated meta-analysis and studies in the original meta-analysis by Bouthoorn et al. The combined prevalence of our and Bouthoorn et al’s meta-analyses is shown in bold black font. This figure is available as part of a downloadable slideset

The prevalence of LVSD (reported in *n*=8 studies; based on a total of 16,918 individuals), yielded a summary prevalence of 12% (95% CI 9%, 17%) for individuals from the general population (*n*=2 studies, with a medium risk of bias) and 3% (95% CI 1%, 12%) for a hospital population (*n*=6 studies, 5/6 of which had a medium risk of bias and 1/6 had a high risk of bias). Overall, studies showed a high level of heterogeneity (*I*^2^=77–94%). Our findings are in contrast to the estimates reported by Bouthoorn et al [12], who found that, on average, 2% (95% CI 2%, 3%) of the general population and 18% (95% CI 17%, 19%) of the hospital population had LVSD. Compared to Bouthoorn et al, we used a different method to statistically handle the occurrence of 0% prevalences in individual studies (Freeman-Turkey transformation in the paper by Bouthoorn et al vs continuity correction in our analysis [‘Systematic review and meta-analysis’ section of the ESM Methods]). Both methods lead to different summary estimates: 18% (95% CI 17%, 19%) found by Bouthoorn et al vs 8% (95% CI 3%, 19%) in our analysis for the hospital population (Fig. 3, section Hospital population [Bouthoorn]), and 2% (95% CI 2%, 3%) found by Bouthoorn et al vs 3% (95% CI 1%, 7%) in our analysis for the general population (Fig. 3, section General population [Bouthoorn]). The use of different, albeit valid, methods can partly explain the difference in found prevalence estimates. Furthermore, our updated analysis in the general population included two studies only, both reporting a relatively high prevalence of 10% and 16%. The overall meta-analysis of studies identified by the current review and the meta-analysis by Bouthoorn et al (based on a total of 24,460 individuals from *n*=25 studies) yielded a combined prevalence of 6% (95% CI 3%, 10%) (Fig. 3).

**Fig. 4:**
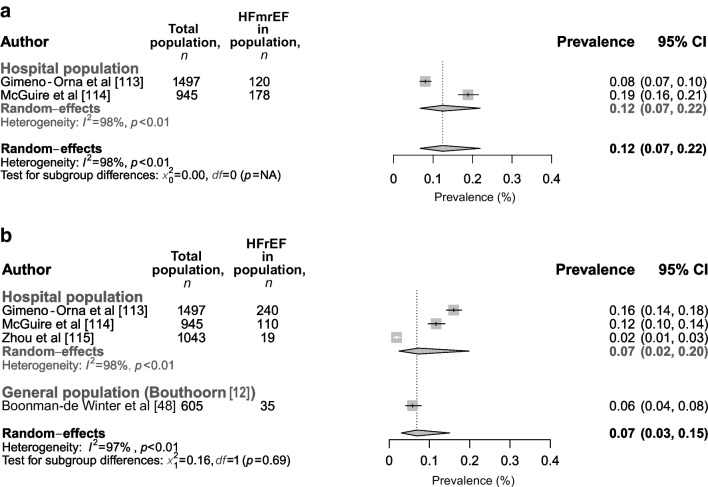
Prevalence of (**a**) HFmrEF and (**b**) HFrEF in individuals with type 2 diabetes in the hospital and general population. For HFrEF results are categorised by new studies included in this updated meta-analysis and studies in the original meta-analysis by Bouthoorn et al. The combined prevalence of our and Bouthoorn et al’s meta-analyses is shown in bold black font. NA, not applicable. This figure is available as part of a downloadable slideset

The prevalence of HFmrEF and HFrEF (reported in *n*=2 and *n*=3 studies, respectively; based on a total of 2442–3485 individuals), yielded a summary prevalence for the hospital population of 12% (95% CI 7%, 22%) for HFmrEF and 7% (95% CI 2%, 20%) for HFrEF (Fig. 4a,b). Overall, the included studies showed a high level of study heterogeneity (*I*^2^=98% for HFmrEF and 98% for HFrEF) and a low–medium level of bias. In the meta-analysis by Bouthoorn et al [12], only one study was included that reported on HFrEF prevalence in the general population (stated as 5.8% [95% CI 3.9%, 7.6%]), which is comparable to our findings. No studies reporting on HFmrEF were identified by the meta-analysis by Bouthoorn et al [12]. For HFrEF, when combining both our and Bouthoorn et al’s meta-analyses (based on a total of 4090 individuals from *n*=4 studies), a prevalence of 7% (95% CI 3%, 15%) was found in the overall population (Fig. 4b).

### LVDD and HFpEF

**Fig. 5:**
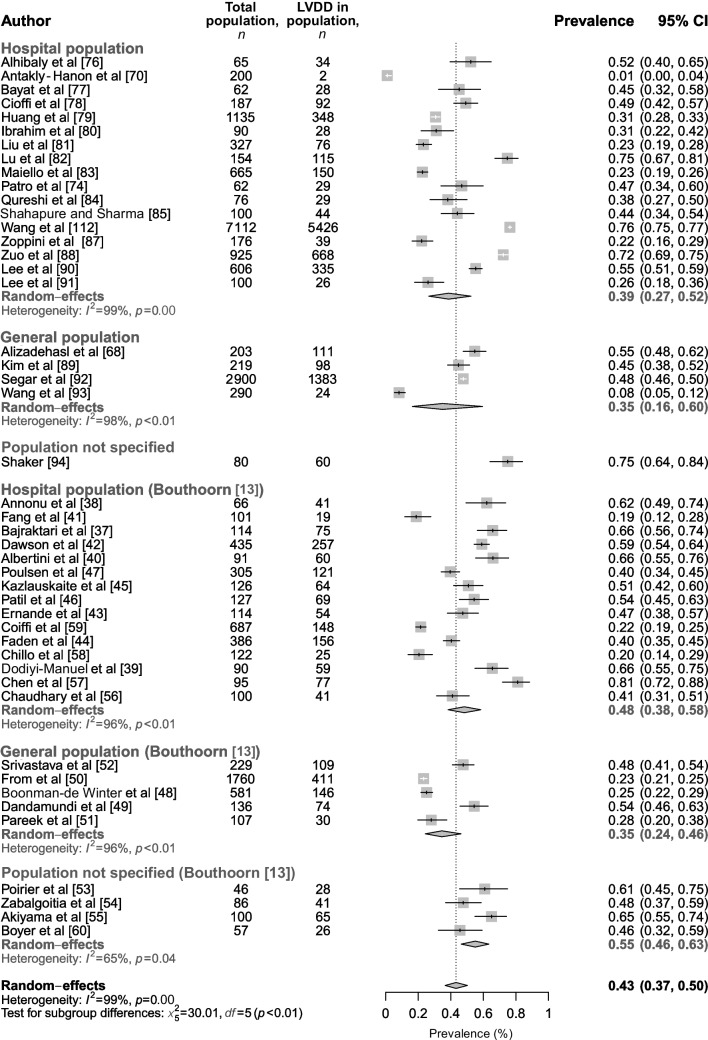
Prevalence of LVDD in individuals with type 2 diabetes in the hospital population, general population and in populations that were not specified. Outcomes are categorised by new studies included in this updated meta-analysis and studies in the original meta-analysis by Bouthoorn et al. The combined prevalence of our and Bouthoorn et al’s meta-analyses is shown in bold black font. This figure is available as part of a downloadable slideset

The analysis of the prevalence of LVDD included 65 studies, and was based on a total of 25,729 individuals. Studies were categorised based on their method of reporting LVDD (binary or categorical). In studies that reported LVDD as a binary variable (yes/no, based on ≥2 echocardiographic parameters), we found an overall prevalence of 38% (95% CI 28%, 49%), of which 39% (95% CI 27%, 52%) were in the hospital population and 35% (95% CI 16%, 60%) were in the general population (Fig. 5). Heterogeneity was high (*I*^2^=97-99%), as was the risk of bias for most studies (10/21), with 9/21 studies scoring a medium risk, one study scoring a low risk of bias, and one for which risk of bias could not be assessed due to unavailability of the supplementary materials. A sensitivity analysis only including studies with an LVEF ≥50% (*n*=10) showed a similar, hence slightly lower prevalence (nominal difference; total population: 27% [95% CI 13%, 47%], *I*^2^=99%; hospital population: 29% [95% CI 13%, 53%], *I*^2^=99%), corresponding to the stricter cut-off value used (ESM Fig. 1). Our findings are comparable with the findings of Bouthoorn et al [13] who reported an LVDD prevalence of 48% (95% CI 38%, 59%) in the hospital population and 35% (95% CI 24%, 46%) in the general population. When all studies were combined (based on a total of 21,795 individuals from *n*=46 studies), an LVDD prevalence of 43% (95% CI 37%, 50%) was found in the total population (Fig. 5).

**Fig. 6:**
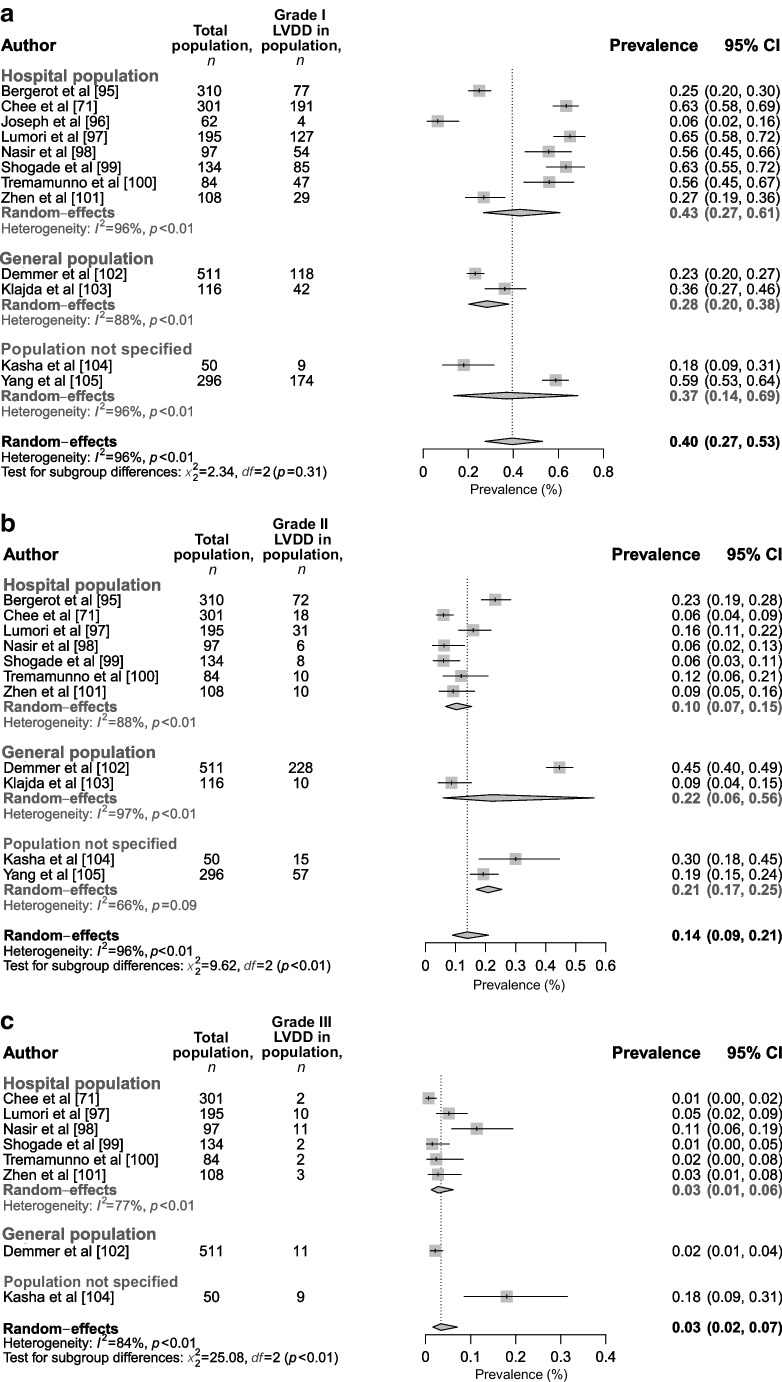
Prevalence of LVDD in individuals with type 2 diabetes in the hospital, general population and in populations that were not specified, categorised as (**a**) grade I, (**b**) grade II and (**c**) grade III based on ASE/EACVI recommendations. This figure is available as part of a downloadable slideset

**Fig. 7:**
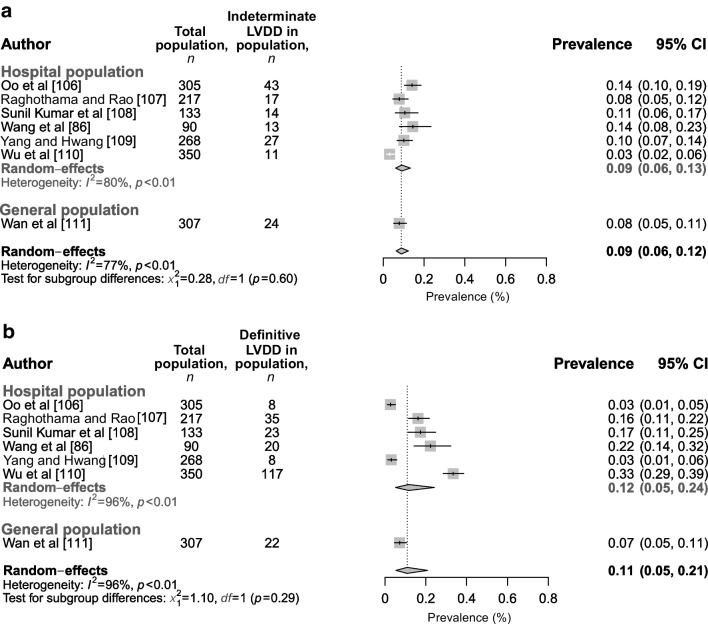
Prevalence of LVDD in individuals with type 2 diabetes in the hospital and general population, categorised as (**a**) indeterminate or (**b**) definitive based on ASE/EACVI recommendations. This figure is available as part of a downloadable slideset

Additionally, we included a large number of studies that reported grade of LVDD (grade I, II or III) (Fig. 6a–c) and/or categories of indeterminate LVDD and definitive LVDD (Fig. 7a,b) according to the ASC/EACVI recommendations [25]. We found that, on average, 40% (95% CI 27%, 53%) of the type 2 diabetes population had grade I LVDD (43% [95% CI 27%, 61%] in a hospital-specific population) based on a total of 2264 individuals, 14% (95% CI 9%, 21%) had grade II LVDD (10% [95% CI 7%, 15%] in a hospital-specific population) based on a total of 2202 individuals, and 3% (95% CI 2%, 7%) had grade III LVDD (3% [95% CI 1%, 6%] in a hospital-specific population) based on a total of 1480 individuals. Heterogeneity was the same for grade I and grade II LVDD (*I*^2^=96%) but appeared better for grade III LVDD (*I*^2^=84%), and most studies showed a low level of bias (7/12), with 2/12 studies showing a medium level of bias and 3/12 studies showing a high level of bias. Sensitivity analysis including studies with an LVEF ≥50% showed similar results (ESM figure 2a–c). Furthermore, when categorised according to indeterminate LVDD vs definitive LVDD, 9% (95% CI 6%, 12%) had indeterminate LVDD (9% [95% CI 6%, 13%] in the hospital population) and 11% (95%CI 5%, 21%) had definitive LVDD (12% [95% CI 5%, 24%] in the hospital population) (Fig. 7), both based on a total of 1670 individuals. Heterogeneity was moderate–high for both the analysis on indeterminate LVDD (*I*^2^=77%) and definitive LVDD (*I*^2^=96%). Sensitivity analysis only including studies with an LVEF ≥50% showed similar results (ESM Fig. 3a,b).

**Fig. 8:**
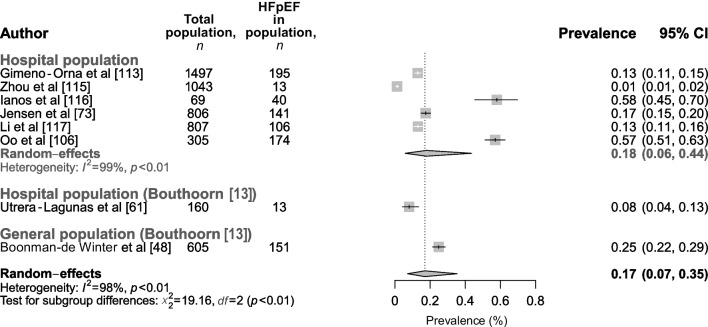
Prevalence of HFpEF in individuals with type 2 diabetes in the hospital and general population. Results are categorised by new studies included in this updated meta-analysis and studies in the original meta-analysis by Bouthoorn et al. The combined prevalence of our and Bouthoorn et al’s meta-analyses is shown in bold black font. This figure is available as part of a downloadable slideset

The analysis of the prevalence of HFpEF included six studies, and was based on a total of 4527 individuals, all of whom were in a hospital population, yielding a summary prevalence of 18% (95% CI 6%, 44%). Overall, studies showed a high level of heterogeneity (*I*^2^=99%). Two studies had a low level of bias and four had a medium level of bias. In the meta-analysis by Bouthoorn et al [13], only two studies were included that reported on HFpEF prevalence, with values ranging from 8% to 25%, which are comparable to our findings. When both our and Bouthoorn et al’s meta-analyses were combined (based on a total of 5292 individuals from *n*=8 studies), a prevalence of 17% (95% CI 7%, 35%) was found for HFpEF in the total population (Fig. 8).

To our knowledge, our review is the first to provide summary estimates of LVDD subcategories among individuals with type 2 diabetes, which give a more accurate reflection of the degree to which this population is affected by LVDD. This is especially important since grade I LVDD is often seen as part of ageing and is not considered clinically relevant. Knowledge on the prevalence of grade II and III LVDD gives more insight into the clinically relevant group of individuals with LVDD, and brings nuance to the high prevalence reported for LVDD in studies that do not report categories of LVDD.

### Discussion of systematic review findings

In general, our findings are in agreement with the findings presented by Bouthoorn et al [12, 13] in their meta-analyses. When both our results and those of Bouthoorn et al were combined, they showed an overall prevalence of 43% for LVDD and 17% for HFpEF, which was much more than the 6% prevalence found for LVSD and 7% prevalence found for HFrEF. In addition, we were able to analyse the prevalence of the different categories of LVDD, bringing more insight into the clinically relevant groups (stage II and III) of individuals with LVDD. Nevertheless, it needs to be acknowledged that moderate–high heterogeneity was present in all analyses. Multiple explanations for the observed heterogeneity can be given: first, slight differences in study design between studies, such as population (e.g., general population vs general hospital population [outpatient and hospital-ward population] vs endocrinology ward population vs cardiology ward population), as well as variation in inclusion and exclusion criteria (e.g., the inclusion or exclusion of people with known ischaemic disease) can result in different estimates. Second, heterogeneity can be introduced due to factors that are inherently connected to the pathophysiology of HF, for example mean age, male/female ratio, diabetes duration and the number of comorbidities for individuals in a subpopulation, since all of these factors are also confounders or mediators in the pathophysiology of HF. Finally, an important cause of heterogeneity is induced by the lack of consensus on methods for diagnosing HF and left ventricular dysfunction (especially for diagnosing LVDD and HFpEF). As mentioned previously, over the past decades, heterogeneous methods and criteria have been used to diagnose LVDD and HFpEF, resulting in heterogeneity in the way in which they have been diagnosed between studies (ESM Tables 3 and 4), which can be seen as a limitation of this analysis. Future studies focusing on subgroups of people with type 2 diabetes, for example female participants only or people with a history of CVD, are needed to map the prevalence of HF in these subpopulations to a further extent. As a final limitation of this study, it should be noted that data extraction and risk-of-bias assessment was performed in twofold for 25% of the studies included (the remaining 75% was done by one author [AGH]); however, good agreement was observed.

## Screening for ventricular dysfunction and HF

Different sets of comorbidities and risk factors have been associated with the development of HFrEF and HFpEF. Along with the direct detrimental effects of hyperinsulinaemia and hyperglycaemia [118], HFrEF most often occurs secondary to comorbidities such as coronary artery disease, chronic kidney disease and hypertension [119–121]. On the other hand, HFpEF is associated with arterial and pulmonary hypertension, obesity and atrial fibrillation, together with multiple pathophysiological mechanisms related to hyperglycaemia, insulin resistance and hyperinsulinaemia [122]. Given that individuals with HF can reside in a pre-clinical phase for years, the question can be raised as to whether screening for HF in a subpopulation can be beneficial.

In a study by van Giessen et al [123], the cost-effectiveness of five screening strategies was investigated; these methods varied from screening medical records to allowing individuals to undergo full echocardiographic screening. The authors found that screening for HF by checking electronic medical records for patient characteristics and medical history plus the assessment of symptoms in patients with type 2 diabetes who were 60 years or older was cost-effective at the commonly used willingness-to-pay threshold of €20,000/quality-adjusted life year (QALY). These findings had a sensitivity of up to 85% and 92% for individuals in the New York Heart Association (NYHA) grading of symptoms for heart failure classifications 2 and 3, respectively, and a specificity of 61% [123]. Echocardiographic screening showed a high effectiveness but at the cost of a higher willingness-to-pay value. To our knowledge, this is the only study of its kind investigating a population with type 2 diabetes. However, screening of patient records and symptoms is invasive, and avoiders of care will likely be missed in this approach. Nevertheless, the authors state that cost-effectiveness increases with increasing effectiveness of therapies [123]. Since this study was conducted before use of SGLT2 inhibitors became standard practice in type 2 diabetes care (which, to date, is the only effective therapy with proven glucose-lowering and cardiovascular-protective effects [15, 16]) screening is likely to be even more beneficial in the present day. Unfortunately, a minimally invasive but sensitive screening tool is lacking. Even though the usefulness and significance of biomarkers for HFrEF in the general population has been established, only NPs and urine albumin/creatinine ratio have been associated with the presence of HFpEF [23, 124, 125] and, as discussed above, these have low specificity and a poor positive-predictive value. Studies investigating the use of NPs to screen for HF have been able to identify over a third of true HF patients; however, they did not make a distinction between HF subtypes in the diagnosis [126, 127]. Furthermore, as aforementioned, NPs tend to increase in the older population, relate inversely to BMI and are affected by kidney function. Overall, even though they are helpful, NPs are not the ideal biomarker where screening is concerned. The majority of studies investigating (novel) diagnostic HF biomarkers, especially those for HFpEF, show a high risk of bias, reducing their reproducibility and the potential for application of their findings in clinical care [128]. A previous review identified emerging biomarkers, including high-sensitivity C-reactive protein (hs-CRP), high-sensitive cardiac troponin T (hs-cTnT) and galectin-3 as possible screening tools for HF [129], and a recently published study found that circulating endotrophin levels are increased in patients with HFpEF and are independently associated with worse outcomes [130]. Nevertheless, more research is needed on the sensitivity on these biomarkers before they can be implemented in clinical practice.

## Incidence of HF in type 2 diabetes

**Fig. 9:**
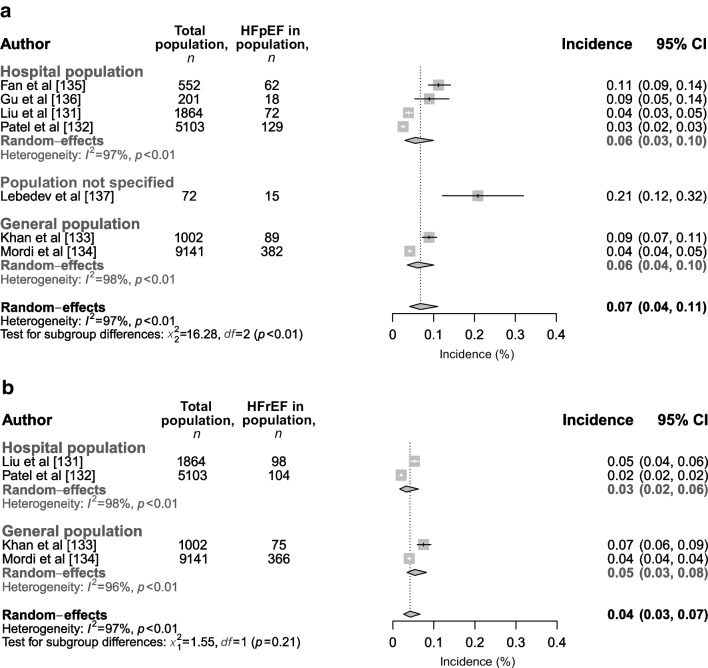
Incidence of (**a**) HFpEF and (**b**) HFrEF in individuals with type 2 diabetes in the hospital population, general population and in populations that were not specified. This figure is available as part of a downloadable slideset

To our knowledge, seven studies (including *n*=17,935 individuals) have separately reported on the incidences of HFpEF [131–137] and HFrEF [131–134] (Table 2), with a follow-up range of 3 to 12.4 years. For HFpEF, cumulative incidences ranged from 2.5% to 20.8% (2.0 to 69.4 cases/1000 person-years) in the hospital population and from 4.2% to 8.9% (4.5 to 7.8 cases/1000 person-years) in the general population. For HFrEF, reported cumulative incidences ranged from 2.0% to 5.3% (1.6 to 7.4 cases/1000 person-years) in the hospital population and from 4.0% to 7.5% (4.3 to 6.6 cases/1000 person-years) in the general population (Table 2). When meta-analysed, a combined overall incidence of 7% (95% CI 4%, 11%) (6% [95% CI 3%, 10%] in the hospital population) and 4% (95% CI 3%, 7%) (3% [95% CI 2%, 6%] in the hospital population) was found for HFpEF and HFrEF, respectively (Fig. 9a,b). Similar to the studies that were included in the meta-analysis for HF prevalence, there was a large variety of methods used to diagnose the HF entities in the studies reporting HF incidence (with the exception that all studies included clinical symptoms as a diagnostic criteria) (see Table 2, ‘Outcome’ column). No studies reporting on the incidence of HFmrEF were found. Our outcomes are comparable with a meta-analysis investigating the overall incidence of HF (not reporting subtypes) in people with type 2 diabetes [138], which found a mean cumulative incidence of overall HF of 10.7% (range: 1.4–39%).

**Table 2:** Incidence of cardiac dysfunction in individuals with type 2 diabetes

Authors (year of publication)	Source population and setting	Age of population, years^a^	Participants with T2D, *n* (% male)	T2D duration, years^a^	Exclusion criteria	Outcome	Follow-up, years^b^	Cumulative incidence rate, %	Incidence rate, cases/1000 person-years
Fan et al (2022) [135]^c^	Hospital setting; individuals with concomitant T2D, hypertension and AF	69.6±7.6	552 (56.9%)	8.2±2.7	LVEF <50%, symptomatic HF, CVD, liver and kidney dysfunction	HFpEF according to 2016 ESC HF criteria	5	11.2 (62/552)	22.5
Gu et al (2018) [136]^c^	Hospital setting; individuals with both T2D and hypertension	65.3±7.5	201 (60.2%)	6.9±2.6 for individuals with low HbA_1c_; 7.4±2.6 for individuals with high HbA_1c_^d^	LVEF <50%, symptomatic HF, CVD, liver and kidney dysfunction	HFpEF according to AHA/ACC diagnostic criteria	7.3	9.0 (18/201)	12.3
Khan et al (2019) [133]	The Health ABC study; community dwelling adults	73.6±2.9	1002 (51.7%)	Not reported	HF at baseline or missing data on CVD	HF based on clinical diagnosis and treatment and EF	11.4 (for entire population)	HFpEF: 8.9 (89/1002); HFrEF: 7.5 (75/1002); unclassified: 5.0% (50/1002)	HFpEF: 7.8; HFrEF: 6.6; unclassified: 4.4
Lebedev et al (2021) [137]	Setting unknown, research centre population; individuals with obesity and T2D	57.0 (49.7–63.2)	72 (55.6%)	8.0 (4.7–12.2)	History of cardiac events	HFpEF, method of diagnosis unknown	3	20.8 (15/72)	69.4
Liu et al (2022) [131]	Outpatient endocrinology clinic	26.6±10.1	1864 (51.0%)	10 (5–16)	History of HF, pregnancy, HbA_1c_ >97 mmol/mol (12%), autoimmune, malignant or end-organ disease	HFpEF according to 2016 ESC HF criteria; HFrEF ≤40%; HFpEF >50%	7.1	HFpEF: 3.9 (72/1864); HFrEF: 5.3 (98/1864)	HFpEF: 5.4; HFrEF: 7.4
Mordi et al (2020) [134]	GoDARTS study; community dwelling individuals who underwent an echocardiogram for clinical reason	65±12	9141 (55.4%)	3.2 (2.2–4.4) for people without MVD; 7.7 (3.2–12.2) for people with MVD	Hospitalisation for HF	HF based on clinical diagnosis and treatment plus EF ≥50% for HFpEF and <50% for HFrEF	9.3	HFpEF: 4.2 (382/9141); HFrEF: 4.0 (366/9141); unclassified: 1.2 (109/9141)	HFpEF: 4.5; HFrEF: 4.3; unclassified: 1.3
Patel et al (2020) [132]	Hospital setting (RCT)	Categorised (mean 30.8–54.1)	5103 (40.3%)	Categorised (mean 6.5–7.1)	HF at baseline, missing data on EF, atherosclerotic disease	Hospitalisation for HF plus EF ≥50% for HFpEF and <50% for HFrEF	12.4	HFpEF: 2.5 (129/5103); HFrEF: 2.0 (104/5103); unclassified: 0.5 (24/5103)	HFpEF: 2.0; HFrEF: 1.6; unclassified: 0.4

^a^Data presented as mean±SD or median (IQR), unless stated otherwise

^b^Data presented as median

^c^Studies from the same overall population but reporting incidences on different subgroups

^d^Categorised on median value of HbA_1c_ variability index: low HbA_1c_, ≤0.66%; high HbA_1c_, >0.66%

ABC, Aging and Body Composition; AF, atrial fibrillation; AHA/ACC, American Heart Association/American College of Cardiology; EF, ejection fraction; GoDARTS, Genetics of Diabetes Audit and Research in Tayside Scotland; MVD, microvascular disease; T2D, type 2 diabetes

## Prognosis of ventricular dysfunction and HF in type 2 diabetes

A substantially increased risk for all-cause mortality, CVD-attributable mortality and first hospitalisation for worsening of HF are observed in individuals with concomitant type 2 diabetes and HF [139–141]. Moreover, for both HFrEF and HFpEF, patients with type 2 diabetes represent a specific clinical phenotype with worse outcomes as compared with patients without type 2 diabetes [142–144]. Individuals with HFpEF and concomitant diabetes who were enrolled in the RELAX trial had a higher risk of hospitalisation for HF compared with those without diabetes (47% vs 28%), as well as a higher risk of hospitalisation for cardiac or renal causes (23.7% vs 4.9%) at 6 months after enrolment [142]. Using data from the Get With the Guidelines Heart Failure registry for patients with HFpEF hospitalised for new or worsening HF, type 2 diabetes was associated with a significantly longer length of stay (OR 1.27 [95% CI 1.23, 1.31]), a lower likelihood of home discharge (OR 0.83 [95% CI 0.81, 0.86]) and an increased likelihood of all-cause 30-day readmission (HR 1.10 [95% CI 1.05, 1.15]) [143]. Clinical outcomes in the long term were also poorer for these individuals as type 2 diabetes was a significant predictor of risk of all-cause mortality and risk of hospitalisation for HFpEF (HR 1.72 [95% CI 1.1, 2.6]) over a 25±11 month period; this finding was independent of age, BMI, kidney function and functional class [144]. Similar results have been found for HFrEF, whereby, in a number of consecutive trials, individuals with both type 2 diabetes and HFrEF had higher risk of all-cause mortality (HR 1.3–2.0) and CVD-attributable mortality (HR 1.5–1.8) compared with those without type 2 diabetes [145–150].

Limited data are available on the prognosis of HFpEF vs HFrEF in people with type 2 diabetes. A large meta-analysis in the general population showed that the risk of all-cause mortality was significantly lower in individuals with HFmrEF (37.5%) than those with HFrEF (43.7%) and HFpEF (47.3%), and that individuals with HFrEF had a lower risk of all‐cause mortality compared with those with HFpEF (HFpEF vs HFrEF: OR 1.0 [95% CI 1.0, 1.1]; *p*=0.01). CVD-attributable mortality was lowest in individuals with HFpEF (11.4%), and highest in those with HFrEF (21.1%), mainly owing to HF-associated death and sudden cardiac death. In comparison, a subgroup analysis in individuals with type 2 diabetes showed that the risk of all‐cause mortality in this population followed a contrasting pattern to that in the general population, with the highest risk of mortality being found for HFpEF and the lowest risk for HFrEF [151]. However, only two studies with contrasting results were included in this subgroup analysis and statistical significance was not reached [151]. Therefore, no conclusions can be made regarding the risk of all-cause and CVD-attributable mortality in individuals with HFpEF vs those with HFrEF.

## Conclusion and future directions

HF and type 2 diabetes are two highly intertwined diseases and concomitantly pose an increased risk of morbidity and mortality. Early diagnosis and treatment might delay clinical progression and, therefore, a timely diagnosis of HF and identification of people at risk for HF is important, with epidemiological knowledge being an essential tool in this process. In this review, we aimed to shed a light on these aspects of HF in individuals with type 2 diabetes.

This updated meta-analysis and the studies by Bouthoorn et al [12, 13] showed an overall prevalence of 43% (95% CI 37%, 50%) and 17% (95% CI 7%, 35%) for LVDD and HFpEF respectively, and a prevalence of 6% (95% CI 3%, 10%) and 7% (95% CI 3%, 15%) for LVSD and HFrEF, respectively, hereby establishing that LVDD and HFpEF are more prevalent in type 2 diabetes than the other forms of HF and LVSD. Furthermore, we reported a higher incidence rate of HFpEF than HFrEF (7% [95% CI 4%, 11%] vs 4% [95% CI 3%, 7%]). In an additional analysis, for LVDD, we found that grade I and/or indeterminate function were highly prevalent and likely to be responsible for the high overall LVDD prevalence rates reported. It must be noted, however, that mild diastolic abnormalities (that place people in grade I/indeterminate function categories of LVDD) are often seen as part of ageing. Overall, these findings suggest that there is a large pre-clinical target group that has early LVDD in which disease progression could be halted by early recognition and adequate treatment, thereby reducing disease burden.

Moving forward, we believe there is need for easily accessible and reliable tools for diagnosing HF. Even though NPs are widely incorporated in practice, they are not optimal for screening and diagnosing HFpEF. The cost–benefit of screening is proven, and the cost-effectiveness increases with increasing effectiveness of therapies, such as SGLT2 inhibitors, therefore thorough clinical investigation is recommended when HF is suspected. The large heterogeneity shown in the studies included in this review can be minimalised by having one uniform way of diagnosing (pre-)clinical entities of HF. With the development of new, uniform and clinically accessible ESC 2021 guidelines for diagnosing HF (especially LVDD/HFpEF), we hope that a consensus is reached about the best way to report subtypes of HF, leading to less heterogeneity in future studies. This will lead to more accurate HF diagnosis, more reliable data and more reliable tools to measure change/progression of HF. When combined, this will ultimately lead to more knowledge and better care for patients with type 2 diabetes and HF.

### Supplementary Information

Below is the link to the electronic supplementary material.

Supplementary file1 (PDF 1.14 MB)

Supplementary file2 (PPTX 1.43 MB)

## Data Availability

The data that support the findings of this study were extracted from the cited papers and can be found in Table 1. R scripts and raw study material are available from the corresponding author upon reasonable request.

## Abbreviations

ASE: American Society of Echocardiography
EACVI: European Association of Cardiovascular Imaging
ESC: European Society of Cardiology
HF: Heart failure
HFA-PEFF: Heart Failure Association Pre-test assessment, Echocardiography and natriuretic peptide, Functional testing, Final etiology
HFmrEF: Heart failure with mildly reduced ejection fraction
HFpEF: Heart failure with preserved ejection fraction
HFrEF: Heart failure with reduced ejection fraction
LVDD: Left ventricular diastolic dysfunction
LVEF: Left ventricle ejection fraction
LVSD: Left ventricular systolic dysfunction
NP: Natriuretic peptides
PRISMA: Preferred Reporting Items for Systematic Reviews and Meta-Analyses
SGLT2: sodium−glucose cotransporter 2
