# Supplementary material for: Epidemiology of heart failure in diabetes: a disease in disguise
Source: Diabetologia. 2024 Feb 9;67(4):574–601. doi: 10.1007/s00125-023-06068-2 (PMC10904471; doi:10.1007/s00125-023-06068-2)
Supplement: Supplementary file 1 — Supplementary file1 (PDF 1.14 MB) [file 125_2023_6068_MOESM1_ESM.pdf]

ESM Methods: Search strategies

Embase
**'heart failure'**/exp OR (**'heart failure'**:ab,ti AND (**'diastolic'**:ab,ti OR **'systolic'**:ab,ti)) OR ‘ejection fraction’:ab,ti OR **'heart ventricle function'**/exp OR (**'failure'**:ab,ti OR **'decompensation'**:ab,ti OR **'insufficiency'**:ab,ti OR **'dysfunction'**:ab,ti OR **'disfunction'**:ab,ti AND (**'ventricular'**:ab,ti OR **'cardiac'**:ab,ti OR **'heart'**:ab,ti OR **'myocardial'**:ab,ti)) AND (**'non insulin dependent diabetes mellitus'**/exp OR **'diabetes mellitus'**:ab,ti OR **'t2d'**:ab,ti) AND (**'prevalence'**/exp OR **'prevalence'**:ab,ti OR **'incidence'**/exp OR **'incidence'**:ab,ti OR **'occurrence'**:ab,ti OR **'frequency'**:ab,ti OR **'rate'**:ab,ti OR **'rates'**:ab,ti OR **'frequencies'**:ab,ti OR **'percentage'**:ab,ti OR **'percentages'**:ab,ti OR **'hf ref'**:ab,ti OR **'hf pef'**:ab,ti) AND [embase]/lim NOT [medline]/lim AND ([dutch]/lim OR [english]/lim) AND (**'article'**/it OR **'article in press'**/it OR **'review'**/it)<br><br>AND<br><br>'heart failure'/exp OR ('heart failure':ab,ti AND ('diastolic':ab,ti OR 'systolic':ab,ti)) OR 'heart ventricle function'/exp OR ('failure':ab,ti OR 'decompensation':ab,ti OR 'insufficiency':ab,ti OR 'dysfunction':ab,ti OR 'disfunction':ab,ti AND ('ventricular':ab,ti OR 'cardiac':ab,ti OR 'heart':ab,ti OR 'myocardial':ab,ti)) AND ('non insulin dependent diabetes mellitus'/exp OR 'diabetes mellitus':ab,ti OR 't2d':ab,ti) AND ('prevalence'/exp OR 'prevalence':ab,ti OR 'incidence'/exp OR 'incidence':ab,ti OR 'occurrence':ab,ti OR 'frequency':ab,ti OR 'rate':ab,ti OR 'rates':ab,ti OR 'frequencies':ab,ti OR 'percentage':ab,ti OR 'percentages':ab,ti OR 'hf ref':ab,ti OR 'hf pef':ab,ti) AND [embase]/lim NOT [medline]/lim AND ([dutch]/lim OR [english]/lim) AND ('article'/it OR 'article in press'/it OR 'review'/it)

Medline
(((((((("Heart Failure"[Mesh:noexp]) OR ((heart failure[Title/Abstract]) AND ((diastolic[Title/Abstract]) OR systolic[Title/Abstract]))) OR ejection fraction[Title/Abstract] OR "Ventricular Dysfunction"[Mesh]) OR (((((((failure[Title/Abstract]) OR decompensation[Title/Abstract]) OR insufficiency[Title/Abstract]) OR dysfunction[Title/Abstract]) OR disfunction[Title/Abstract])) AND (((ventricular[Title/Abstract]) OR cardiac[Title/Abstract]) OR heart[Title/Abstract] OR myocardial[Title/Abstract])))) AND ((("Diabetes Mellitus, Type 2"[Mesh:noexp]) OR diabetes mellitus[Title/Abstract]) OR T2D [Title/Abstract]))) AND (((("Prevalence"[Mesh]) OR prevalence[Title/Abstract]) OR "Incidence"[Mesh]) OR ((((((((incidence[Title/Abstract]) OR occurence[Title/Abstract]) OR frequency[Title/Abstract]) OR rate[Title/Abstract]) OR rates[Title/Abstract]) OR frequencies[Title/Abstract]) OR percentage[Title/Abstract]) OR percentages[Title/Abstract] OR (Hf ref[Title/Abstract]) OR (Hf pef[Title/Abstract]))))<br><br>AND<br><br>(((((((("Heart Failure"[Mesh:noexp]) OR ((heart failure[Title/Abstract]) AND ((diastolic[Title/Abstract]) OR systolic[Title/Abstract]))) OR "Ventricular Dysfunction"[Mesh]) OR (((((((failure[Title/Abstract]) OR decompensation[Title/Abstract]) OR insufficiency[Title/Abstract]) OR dysfunction[Title/Abstract]) OR disfunction[Title/Abstract])) AND (((ventricular[Title/Abstract]) OR cardiac[Title/Abstract]) OR heart[Title/Abstract] OR myocardial[Title/Abstract])))) AND ((("Diabetes Mellitus, Type 2"[Mesh:noexp]) OR diabetes mellitus[Title/Abstract]) OR T2D [Title/Abstract]))) AND (((("Prevalence"[Mesh]) OR prevalence[Title/Abstract]) OR "Incidence"[Mesh]) OR ((((((((incidence[Title/Abstract]) OR occurence[Title/Abstract]) OR frequency[Title/Abstract]) OR rate[Title/Abstract]) OR rates[Title/Abstract]) OR frequencies[Title/Abstract]) OR percentage[Title/Abstract]) OR percentages[Title/Abstract] OR (Hf ref[Title/Abstract]) OR (Hf pef[Title/Abstract]))))

ESM Methods: Systematic review and meta-analysis

Search strategy, study selection, data extraction and quality assessment

We conducted a systematic search in Medline and Embase for papers published from 2016 to 20 October 2022. Since our review is an update of the reviews published by Bouthoorn et al. (1, 2) we used a comparable search strategy (ESM Methods: Search strategies). The protocol for this review was registered in the International Prospective Register of Systematic Reviews, the PROSPERO database, under number: CRD42022368035 (https://www.crd.york.ac.uk/prospero/display_record.php?RecordID=368035).

Studies published in English and Dutch were considered. Letters, editorials, case reports, practical guidelines and animal or laboratory studies were excluded. Studies using data from the population at large, as well as hospital populations were included, but reported and analyzed separately. If multiple studies were based on the same study population, we selected the study with the largest population for data extraction.

Definition of study outcome

1. Prevalence of left ventricular diastolic dysfunction in people with type 2 diabetes; Only studies that used echocardiography to establish or confirm the diagnosis are included.
    a. LVDD was defined as an ejection fraction of ⩾45-50% and diastolic abnormalities on echocardiography such as an E/A ratio < 0.75 or >1.50, E/e' ratio > 13 and left atrial (LA) volume index > 34 mL/m2.
2. Prevalence of HFpEF in people with type 2 diabetes; Only studies that used echocardiography to establish or confirm the diagnosis are included.
    a. HFpEF was defined as having an ejection fraction of ⩾45-50% and clinical symptoms and signs suggestive of HF (i.e. shortness of breath, fatigue, pulmonary congestion and/or peripheral edema) and objective evidence of diastolic dysfunction measured with echocardiography
3. Prevalence of left ventricular systolic dysfunction in people with type 2 diabetes; Only studies that used echocardiography to establish or confirm the diagnosis are included.
    a. LVSD was defined as an ejection fraction of <50% and systolic abnormalities on echocardiography.
4. Prevalence of HFrEF in people with type 2 diabetes; Only studies that used echocardiography to establish or confirm the diagnosis are included.
    a. HFrEF was defined as having an ejection fraction of <50% and clinical symptoms and signs suggestive of HF (i.e. shortness of breath, fatigue, pulmonary congestion and/or peripheral edema)

Participants/population

1. People with type 2 diabetes, male and female ≥ 18 years old
    a. defined by one of the following criteria: documentation in medical record, physician's diagnosis, self-reported history, use of anti-diabetic agents and random serum glucose ⩾ 200 mg/dL (or ⩾11.1 mmol/L) or serum fasting glucose ⩾ 126 mg/dL (or ⩾7.0 mmol/L)
2. No restrictions are placed on the study population

Types of study to be included

Observational studies (cross-sectional and prospective studies), and interventional studies reporting (baseline) prevalences of undiagnosed LVDD/LVSD and/or HFpEF/HFrEF in a certain population

Main outcomes

a. Prevalence of undiagnosed LVDD in people with type 2 diabetes; Only studies that used echocardiography to establish or confirm the diagnosis are included.
    i. LVDD was defined as an ejection fraction of ⩾45% and diastolic abnormalities on echocardiography such as an E/A ratio < 0.75 or >1.50, E/é ratio > 13 and left atrial (LA) volume index > 34 mL/m2.
    ii. when LVDD, studies were categorized according to their LVDD output style (1) Grade I/II/III, (2) Indeterminate LVDD / definitive LVDD and analysed separately.
b. Prevalence of undiagnosed HFpEF in people with type 2 diabetes; Only studies that used echocardiography to establish or confirm the diagnosis are included.
    i. HFpEF was defined as having an ejection fraction of ⩾45% and clinical symptoms and signs suggestive of HF (i.e. shortness of breath, fatigue, pulmonary congestion and/or peripheral edema) and objective evidence of diastolic dysfunction measured with echocardiography
c. Prevalence of left ventricular systolic dysfunction in people with type 2 diabetes; Only studies that used echocardiography to establish or confirm the diagnosis are included.
    i. LVSD was defined as an ejection fraction of <50% and systolic abnormalities on echocardiography.
d. Prevalence of HFrEF in people with type 2 diabetes; Only studies that used echocardiography to establish or confirm the diagnosis are included.
    i. HFrEF was defined as having an ejection fraction of <50% and clinical symptoms and signs suggestive of HF (i.e. shortness of breath, fatigue, pulmonary congestion and/or peripheral edema)

From the criteria mentioned above, the following patient, intervention, comparison, outcome (PICO) was obtained:
Population: We included observational studies of adults (age ≥18 years) who underwent echocardiographic measurements either in a research setting in the general population or in a hospital setting, and were not diagnosed with any form of heart failure before inclusion.
Intervention/measurement: Data on patients having ventricular dysfunction, measured by echocardiography (using the cut-offs described above) and the presence of heart failure (and its subtypes (using the cut-offs described above in patients that exert symptoms of heart failure) were considered.
Outcome: To be included (cross sectional) data on the presence and absence of ventricular dysfunction and/or heart failure (LVDD, LVSD, HFpEF, HFrEF and/or HFmrEF) needed to be present and reported. Data was either presented in percentages, or absolute numbers of which percentages were calculated manually.

Initial screening was done by three reviewers (A.G.H; J.W.B.; E.W.), selection was done by two reviewers (A.G.H.; J.W.B.), data extraction was done by A.G.H. and 25% was scored in twofold.

Screening and selection was done independently, and consensus was used to resolve disagreement. There were no automation tools used in the screening and selection process.

In agreement with the reviews published by Bouthoorn et al. (1, 2), a methodological quality assessment (risk of bias assessment) of the included studies was performed (one author, A.G.H.), which was based on the risk of bias tool of Hoy et al. (3), using signaling questions to identify potential problems in the design, conduct and analysis of a study. Signaling questions were scored separately (low or high risk of bias) and an overall score of bias (low: =< 1 points of high risk, medium: 2-3 points of high risk, high: >3 points of high risk) was given to each included study. Studies were assessed by one author (A.G.H.), but a selection (25%) was performed in twofold with an excellent agreement for data extraction (absolute agreement 98%) and a good agreement for risk of bias (absolute agreement on final score: 74%, compared to an expected 72% reported by Hoy et al. in the validation process. No automation tools were used.

Data synthesis and analysis

Data extraction included: first author's name, publication year, study design, study population and population characteristics, relevant selection criteria (in and exclusion), number of participants (%male), age, duration of type 2 diabetes, method of diagnosing HF/ventricular dysfunction, number of patients with LVDD/LVSD and/or HFpEF/HFrEF, total number of participants in study population. Since prevalence estimates are based on cross sectional data, only baseline cross-sectional data was taken into account during the data extraction process.

Individual study prevalence and the corresponding 95% confidence intervals (CIs) were calculated for all included studies. To perform meta-analysis, the prevalence data was logit transformed in order to make the data follow a normal distribution. A transformation is needed to stabilize the variance in a meta-analysis on prevalence data (4). We used the automated tool embedded in the R package 'meta' to perform the (back)transformation. A random-effects model was used to obtain pooled estimates (with the corresponding 95% CI) of the logit-transformed prevalence data, as this model takes the between-study heterogeneity into account better than a fixed-effects model. Continuity correction (0.5%) was used in studies reporting a prevalence of zero (only used to calculate individual study results). Heterogeneity was assessed using the I2 statistic. The pooled prevalence estimate was calculated for all the included studies and separately for studies concerning the general population and hospital population. If we could not recalculate prevalence estimates or if only figures with no absolute numbers were reported, studies were not included in the meta-analysis. Results of the meta-analysis are presented as forest plots showing prevalence proportions with the corresponding 95% CIs for each study and the overall random-effects pooled estimate. All statistical analyses were performed in R using the 'meta' package version 6.5-0 (CRAN - Package meta (r-project.org)).

ESM Table 1: PRISMA Checklist for Manuscripts

Section and Topic	Item #	Checklist item	Location where item is reported
**TITLE**			
Title	1	Identify the report as a systematic review.	Yes, title page
**ABSTRACT**			
Abstract	2	See the PRISMA 2020 for Abstracts checklist.	See abstract check list
**INTRODUCTION**			
Rationale	3	Describe the rationale for the review in the context of existing knowledge.	Yes, 'Introduction' and 'updated systematic review' section
Objectives	4	Provide an explicit statement of the objective(s) or question(s) the review addresses.	Yes, 'Introduction' and 'updated systematic review' section
**METHODS**			
Eligibility criteria	5	Specify the inclusion and exclusion criteria for the review and how studies were grouped for the syntheses.	Yes, supplementary materials (page 1)
Information sources	6	Specify all databases, registers, websites, organisations, reference lists and other sources searched or consulted to identify studies. Specify the date when each source was last searched or consulted.	Yes, supplementary materials (page 1) and manuscript ('Introduction' and 'updated systematic review' section)
Search strategy	7	Present the full search strategies for all databases, registers and websites, including any filters and limits used.	Yes, supplementary materials (page 4)
Selection process	8	Specify the methods used to decide whether a study met the inclusion criteria of the review, including how many reviewers screened each record and each report retrieved, whether they worked independently, and if applicable, details of automation tools used in the process.	Yes, supplementary materials (page 2-3) and 'updated systematic review' section manuscript
Data collection process	9	Specify the methods used to collect data from reports, including how many reviewers collected data from each report, whether they worked independently, any processes for obtaining or confirming data from study investigators, and if applicable, details of automation tools used in the process.	Yes, supplementary materials (page 2-3) and 'updated systematic review' section manuscript
Data items	10a	List and define all outcomes for which data were sought. Specify whether all results that were compatible with each outcome domain in each study were sought (e.g. for all measures, time points, analyses), and if not, the methods used to decide which results to collect.	Yes, supplementary materials (page 2-3)
	10b	List and define all other variables for which data were sought (e.g. participant and intervention characteristics, funding sources). Describe any assumptions made about any missing or unclear information.	Yes, supplementary materials (page 2-3)

Section and Topic	Item #	Checklist item	Location where item is reported
Study risk of bias assessment	11	Specify the methods used to assess risk of bias in the included studies, including details of the tool(s) used, how many reviewers assessed each study and whether they worked independently, and if applicable, details of automation tools used in the process.	Yes, supplementary materials (page 2-3) and 'updated systematic review' section manuscript
Effect measures	12	Specify for each outcome the effect measure(s) (e.g. risk ratio, mean difference) used in the synthesis or presentation of results.	Yes, in methods in both the main paper ('updated systematic review' section) as in supplementary materials (page 2-3) (prevalence/incidence and 95% confidence interval)
Synthesis methods	13a	Describe the processes used to decide which studies were eligible for each synthesis (e.g. tabulating the study intervention characteristics and comparing against the planned groups for each synthesis (item #5)).	N.A.
	13b	Describe any methods required to prepare the data for presentation or synthesis, such as handling of missing summary statistics, or data conversions.	N.A.
	13c	Describe any methods used to tabulate or visually display results of individual studies and syntheses.	Yes, supplementary materials (page 3)
	13d	Describe any methods used to synthesize results and provide a rationale for the choice(s). If meta-analysis was performed, describe the model(s), method(s) to identify the presence and extent of statistical heterogeneity, and software package(s) used.	Yes, supplementary materials (page 3)
	13e	Describe any methods used to explore possible causes of heterogeneity among study results (e.g. subgroup analysis, meta-regression).	Yes, supplementary materials (page 3) and 'updated systematic review' section manuscript
	13f	Describe any sensitivity analyses conducted to assess robustness of the synthesized results.	Yes, page 'updated systematic review' section manuscript
Reporting bias assessment	14	Describe any methods used to assess risk of bias due to missing results in a synthesis (arising from reporting biases).	N.A.
Certainty assessment	15	Describe any methods used to assess certainty (or confidence) in the body of evidence for an outcome.	Yes, page 'updated systematic review' section manuscript
**RESULTS**			
Study selection	16a	Describe the results of the search and selection process, from the number of records identified in the search to the number of studies included in the review, ideally using a flow diagram.	Yes, figure 2 manuscript
	16b	Cite studies that might appear to meet the inclusion criteria, but which were excluded, and explain why they were excluded.	Number and reason of exclusion of studies which were excluded are given, but studies were not

Section and Topic	Item #	Checklist item	Location where item is reported
			cited given the large amount of studies, figure 2
Study characteristics	17	Cite each included study and present its characteristics.	Yes, table 1 and 'updated systematic review' section manuscript
Risk of bias in studies	18	Present assessments of risk of bias for each included study.	Yes, table 1 and 'updated systematic review' section manuscript
Results of individual studies	19	For all outcomes, present, for each study: (a) summary statistics for each group (where appropriate) and (b) an effect estimate and its precision (e.g. confidence/credible interval), ideally using structured tables or plots.	Yes, figure 3-9 and supplementary figures
Results of syntheses	20a	For each synthesis, briefly summarise the characteristics and risk of bias among contributing studies.	Yes, 'updated systematic review' section and table 1
	20b	Present results of all statistical syntheses conducted. If meta-analysis was done, present for each the summary estimate and its precision (e.g. confidence/credible interval) and measures of statistical heterogeneity. If comparing groups, describe the direction of the effect.	Yes, 'updated systematic review' section and figures 3-9
	20c	Present results of all investigations of possible causes of heterogeneity among study results.	Yes, 'updated systematic review' section
	20d	Present results of all sensitivity analyses conducted to assess the robustness of the synthesized results.	Yes, 'updated systematic review' section
Reporting biases	21	Present assessments of risk of bias due to missing results (arising from reporting biases) for each synthesis assessed.	No missing results were present
Certainty of evidence	22	Present assessments of certainty (or confidence) in the body of evidence for each outcome assessed.	Yes, 'updated systematic review' section
**DISCUSSION**			
Discussion	23a	Provide a general interpretation of the results in the context of other evidence.	Yes, 'updated systematic review' section
	23b	Discuss any limitations of the evidence included in the review.	Yes, 'updated systematic review' section
	23c	Discuss any limitations of the review processes used.	Yes, 'updated systematic review' section
	23d	Discuss implications of the results for practice, policy, and future research.	Yes, 'updated systematic review' section
**OTHER INFORMATION**			

Section and Topic	Item #	Checklist item	Location where item is reported
Registration and protocol	24a	Provide registration information for the review, including register name and registration number, or state that the review was not registered.	Yes, abstract and ‘updated systematic review’ section
	24b	Indicate where the review protocol can be accessed, or state that a protocol was not prepared.	Yes, abstract and ‘updated systematic review’ section
	24c	Describe and explain any amendments to information provided at registration or in the protocol.	N.A.
Support	25	Describe sources of financial or non-financial support for the review, and the role of the funders or sponsors in the review.	Yes, ‘Support, relationships and activities’
Competing interests	26	Declare any competing interests of review authors.	Yes, ‘Authors’ relationships and activities’
Availability of data, code and other materials	27	Report which of the following are publicly available and where they can be found: template data collection forms; data extracted from included studies; data used for all analyses; analytic code; any other materials used in the review.	Yes, ‘Data availability’

ESM Table 2: PRISMA checklist for abstracts

Section and Topic	Item #	Checklist item	Reported (Yes/No)
**TITLE**			
Title	1	Identify the report as a systematic review.	Yes
**BACKGROUND**			
Objectives	2	Provide an explicit statement of the main objective(s) or question(s) the review addresses.	Yes
**METHODS**			
Eligibility criteria	3	Specify the inclusion and exclusion criteria for the review.	Yes
Information sources	4	Specify the information sources (e.g. databases, registers) used to identify studies and the date when each was last searched.	Yes
Risk of bias	5	Specify the methods used to assess risk of bias in the included studies.	Yes
Synthesis of results	6	Specify the methods used to present and synthesise results.	Yes
**RESULTS**			
Included studies	7	Give the total number of included studies and participants and summarise relevant characteristics of studies.	Yes
Synthesis of results	8	Present results for main outcomes, preferably indicating the number of included studies and participants for each. If meta-analysis was done, report the summary estimate and confidence/credible interval. If comparing groups, indicate the direction of the effect (i.e. which group is favoured).	Yes
**DISCUSSION**			
Limitations of evidence	9	Provide a brief summary of the limitations of the evidence included in the review (e.g. study risk of bias, inconsistency and imprecision).	Yes
Interpretation	10	Provide a general interpretation of the results and important implications.	Yes
**OTHER**			
Funding	11	Specify the primary source of funding for the review.	Yes
Registration	12	Provide the register name and registration number.	Yes

ESM Table 3: Overview of different methods used to diagnose left ventricular diastolic dysfunction in patients with type II diabetes, included in the systematic review and meta-analysis

NAME	EJECTION FRACTION CUT-OFF USED	METHOD USED TO DIAGNOSE LVDD	REMARKS	CATEGORIZED
BERGEROT (2018) **(5)**	< 55% excluded	2009 ASE/EACVI reccomendations (6)		Normal, grade I, grade II, grade III
CHEE (2021) **(7)**	< 50% excluded	2016 ASE/EACVI reccomendations (8)		Normal, grade I, grade II, grade III
JOSEPH (2020) **(9)**	< 50% considdered systolic dysfunction	2016 ASE/EACVI reccomendations (8)		Normal, grade I, grade II, grade III
LUMORI (2022) **(10)**	Not specified	2016 ASE/EACVI reccomendations (8)		Normal, grade I, grade II, grade III
NASIR (2016) **(11)**	"Normal", not further specified	Presence of: E/a < 1 and/or DT < 160 or >220 and/or E'<8ms		Normal, grade I, grade II, grade III
SHOGADE (2018) **(12)**	Not specified	2009 ASE/EACVI reccomendations (6)		Normal, grade I, grade II, grade III
TREMAMUNNO (2022) **(13)**	Not specified	2016 ASE/EACVI reccomendations (8)		Normal, grade I, grade II, grade III
ZHEN (2016) **(14)**	Not specified	2009 ASE/EACVI reccomendations (6)		Normal, grade I, grade II, grade III
DEMMER (2016) **(15)**	Not specified	Score incorporating the definitions using a combination of 2009 ASE/EACVI reccomendations (6) and Redfield definitions (16)		Normal, grade I, grade II, grade III
KLAJDA (2020) **(17)**	Not specified	Not specified, but referred to the Redfield definitions (16)	normal (0.75 < E/A<1.5 and E/e' < 10); mild (defined as impaired relaxation without increased filling pressures, E/A ≤ 0.75 and E/e' < 10); moderate (defined as impaired relaxation associated with moderately elevated filling pressures or pseudonormal filling, 0.75 < E/A < 1.5 and E/e' ≥ 10); and severe (defined as advanced reduction in compliance or reversible or fixed restrictive filling, E/A > 1.5 and E/e' ≥ 10	Normal, grade I, grade II, grade III
KASHA (2017) **(18)**	<55% excluded	Based on E, A E/A, IVRT, PASP. No specific cut-offs or algorythm mentioned		Normal, grade I, grade II, grade III
YANG (2016) **(19)**	<40% excluded	2009 ASE/EACVI reccomendations (6)		Normal, grade I, grade II, grade III
OO (2021) **(20)**	Not specified	2016 ASE/EACVI reccomendations (8)		Normal, indeterminate, diastolic dysfunction

RAGHOTHAMA (2021) **(21)**	< 50% excluded	2016 ASE/EACVI reccomendations (8)		Normal, indeterminate, diastolic dysfunction
SUNIL KUMAR (2021) **(22)**	< 50% excluded	2016 ASE/EACVI reccomendations (8)		Normal, indeterminate, diastolic dysfunction
WANG (2022) **(23)**	Not specified	2016 ASE/EACVI reccomendations (8)		Normal, indeterminate, diastolic dysfunction
YANG (2022) **(24)**	<40% excluded	2016 ASE/EACVI reccomendations (8)		Normal, indeterminate, diastolic dysfunction
WU (2021) **(25)**	Not specified	2016 ASE/EACVI reccomendations (8)		Normal, indeterminate, diastolic dysfunction
WAN (2019) **(26)**	"normal ejection fraction", not further specified in exclusion criteria	2016 ASE/EACVI reccomendations (8)		Normal, indeterminate, diastolic dysfunction
ALHIBALY (2021) **(27)**	Not specified	2016 ASE/EACVI reccomendations (8)	One category where grades of LVDD are combined	one category
ANTAKLY-HANON (2020) **(28)**	Not specified	2016 ASE/EACVI reccomendations (8)	Presence of at least three of the following: average E/e' >14; septal e' velocity <7 cm/s or lateral e' velocity <10 cm/s; TR velocity 2.8 m/s; LA volume index >34 mL/m2	one category
BAYAT (2020) **(29)**	<50% excluded	Not specified	DT, the S wave, and E and A waves and their ratio (E/A, Ea, and E/Ea). No cut-offs reported	one category
CIOFFI (2021) **(30)**	"normal ejection fraction", not further specified in exclusion criteria	Not specified	E/A, DT, LAVI, PASP are mentioned, no cut-offs or algorythm reported	one category
HUANG (2022) **(31)**	<50% excluded	2016 ASE/EACVI reccomendations (8)	Two of the following criteria were met: (1) E/e' ratio > 14; (2) septal e' < 7 cm/s; (3) TR velocity > 2.8 m/s; (4) LAVi > 34 ml/m	one category
IBRAHIM (2021) **(32)**	Not specified	2016 ASE/EACVI reccomendations (8)	One category where grades of LVDD are combined	one category
LIU (2021) **(33)**	<50% excluded	Roughly based on 2016 ASE/EACVI reccomendations (8)	LVDD at rest was diagnosed if any three or more of the following criteria were met: 1) average E/e’ > 14, 2) septal e’ < 7cm/s or lateral e’ < 10cm/s, 3) TRPV > 2.8m/s, and 4) LAVI > 34mL/m2 .	one category
LU (2017) **(34)**	Not specified	Not specified	Not specified	one category
MAIELLO (2017) **(35)**	≤45% excluded	2009 ASE/EACVI reccomendations (6)	One category where grades of LVDD are combined	one category
PATRO (2021) **(36)**	Not specified	Not specified	Parameters like IVRT, LVEF, DT, Fractional Shortening, Cardiac Output, E/A velocity ratio, LV Internal Diameter Systolic, LV Internal Diameter Diastolic, and E Point Septal Separation were calculated. Not further specified	one category

QURESHI (2016) **(37)**	Not specified	Not specified	E/A ratio of less than 1, mitral DT >240 mitral and IVRT > 90 msec	one category
SHAHAPURE (2020) **(38)**	Not specified	Not specified	E/A (<2), DT (<200 and >160). No specific algorythm mentioned	one category
WANG (2022) **(39)**	Not specified	2016 ASE/EACVI reccomendations (8)	One category where grades of LVDD are combined	one category
ZOPPINI (2018) **(40)**	<50% excluded	Roughly based on 2016 ASE/EACVI reccomendations (8)	LVEDV/BSA (<56) and and E/e' (>8) was considered to be true diastolic dysfunction	one category
ZUO (2019) **)** **(41)**	<50% excluded	Not specified	Peak E/A ratio <1 together with "other measurements"	one category
ALIZADEHASL (2021) **(42)**	Not specified	Not specified	Normal, mild and moderate left ventricular diastolic dysfunction based on E/A, LAVi, Tr velocity, and E'. Method of grading not specified.	one category (mild and moderate combined)
KIM (2019) **(43)**	⩽ 52% for male and ⩽ 54% for female, excluded	Not specified	E/Em >, LAVi>= 34 mL/m2, TDI based on abnormality based on standard deviations. Not further specified	one category
LEE (2020) **(44)**	Not specified	Not specified	E/A <1 or DT >240 ms (age <55 years) or E/A <0.8 and DT >240 ms (age ≥55 years); pseudonormal, E/ A 1 to 1.5 and DT >240 ms; or restrictive, DT <160ms with 1 or more of the following: E/A 1.5 or LA diameter >5 cm.	one category
LEE (2020) **(45)**	Not specified	Not specified	E/e’ >15	one category
SEGAR (2021) **(46)**	<45% excluded	Roughly based on 2016 ASE/EACVI reccomendations (8)	any of the following (as available): 1) E/e >13; 2) E/A <1 and E-wave =<50 cm/s; 3) E/A >2; 4) E/A <1 and pulmonary vein flow reversal (S/D <1); 5) E/A <1 and right ventricle systolic pressure > 35 mm Hg; 6) IVRT >100 ms; or 7) E-wave DT >240 ms	one category
WANG (2018) **(47)**	<40% excluded	Not specified	More than two of the following were present 1) E/e’ was >13, ; 2) LAVi (>34 ml/m2); 3) left ventricular hypertrophy (>115 g/m2 for men, >95 g/m2 for women); and 4) impaired global longitudinal strain (cutoff 16%).	one category
SHAKER (2019) **(48)**	Not specified	Not specified	Not specified	one category

A; atrial contraction wave, ASE/EACVI: American society of echocardiography/European association of cardiovascular imaging, BSA; Body surface area, DT; deceleration time, E; early mitral inflow wave, E/A; early mitral inflow wave / atrial contraction wave, IVRT; isovolumic relaxation time, LA; left atrium, LAVI; left atrial volume index, LV; left ventricle; LVDD; left ventricular diastolic dysfunction; LVEF: left ventricular ejection fraction, PASP; pulmonary artery pressure, TR: tricuspid regurgitation, TDI; tissue doppler imaging

ESM Table 4: Overview of different methods used to diagnose heart failure with preserved ejection fraction in patients with type II diabetes, included in the systematic review and meta-analysis

NAME	EJECTION FRACTION CUT-OFF USED	METHOD USED TO DIAGNOSE HFPEF	CATEGORIZED
GIMENO-ORNA **(49)**	≥40% (≥50% in sensitivity analysis)	2016 ESC guidelines for the diagnosis and treatment of acute and chronic heart failure (50)	Binary
ZHOU **(51)**	>50%	NT-proBNP concentration above the age-specific diagnostic threshold of ≥450pg/mL in age <50 years, ≥900pg/mL in age 50–75 years and ≥1800pg/mL in age >75 years and ejection fraction >50% and LVEF (≥50% to diagnose heart failure with preserved ejection fraction and <50% to diagnose heart failure with reduced ejection fraction)	Binary
IANOS **(52)**	≥50%	HFA-PEFF algorythm (53)	Binary
JENSEN **(54)**	LVEF >40% and ≤50%,	HFpEF was defined as reporting of dyspnea corresponding to the New York Heart Association class II-IV and presence of at least one of the following echocardiographic findings: (a) LVEF >40% and ≤50%, (b) ratio of early diastolic mitral inflow velocity (E) to early diastolic septal annular velocity (e')(E/e´septal) ≥15, (c) increased left ventricular (mass index (>95 g/cm2 for women and >115 g/cm2 for men), and (d) left atrial volume index >34 mL/m2	Binary
LI **(55)**	>50%	HFpEF was diagnosed according to the European Society of Cardiology guideline : (1) presence of symptoms and/or signs of HF; (2) LVEF ≥50 %; (3) NTproBNP >125 pg/mL **(50)**	Binary
OO **(20)**	Not specified	2016 ASE/EACVI recommendations (8), symptoms and NTproBNP values	Binary

ASE/EACVI: American society of echocardiography/European association of cardiovascular imaging, ESC; European society of cardiology, HFpEF; heart failure with preserved ejection fraction, LVEF: left ventricle ejection fraction, NTproBNP; N-terminal pro–B-type natriuretic peptide

ESM Figure 1: Prevalence of LVDD in individuals with type 2 diabetes in the hospital population, general population in studies using a LVEF cut-off of ≥50%. *df*, degrees of freedom.

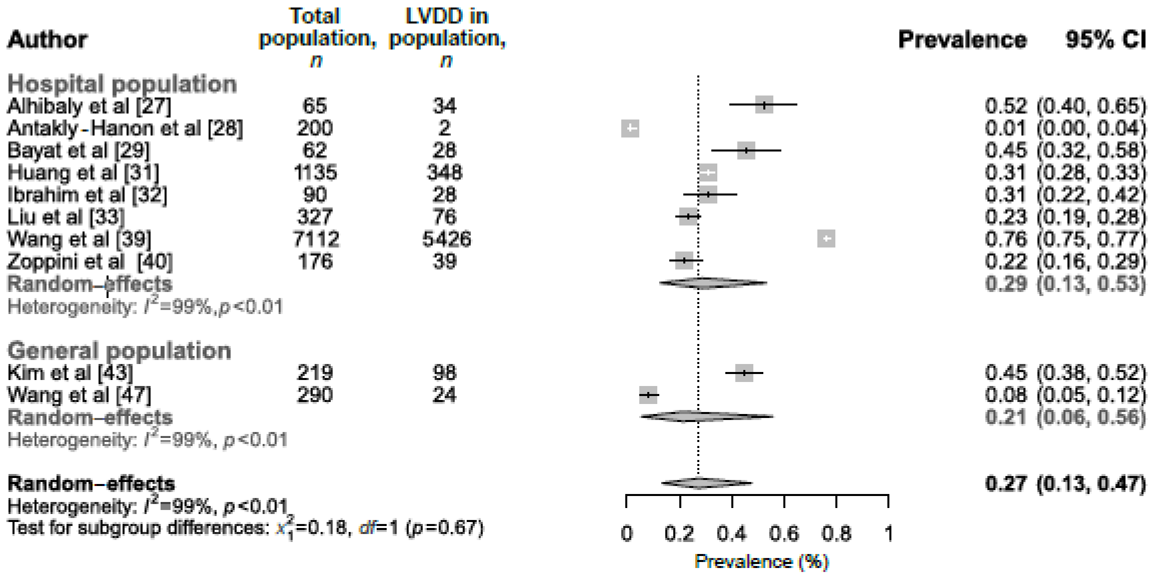

ESM Figure 2: Prevalence of LVDD in individuals with type 2 diabetes in the hospital and general population, categorised as (a) grade I, (b) grade II and (c) grade III based on American society of Echocardiography/European Association of Cardiovascular Imaging (ASE/EACVI) recommendations, in studies using a LVEF cut-off of ≥50%. df, degrees of freedom.

a.

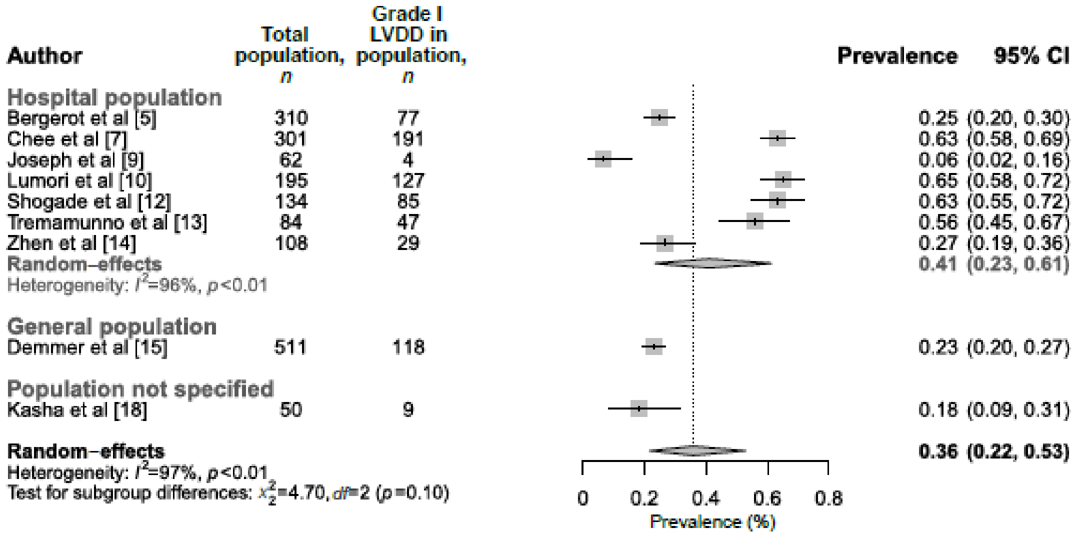

b.

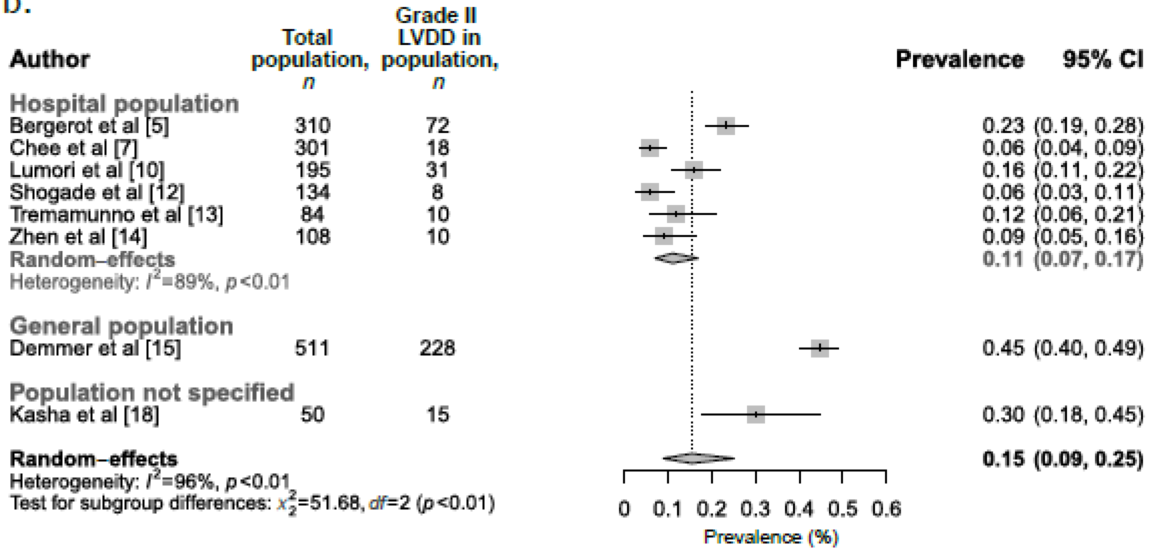
Author
Total population, n
Grade II LVDD in population, n
Prevalence
95% CI
Hospital population
Bergerot et al [5] 310 72 0.23 (0.19, 0.28)
Chee et al [7] 301 18 0.06 (0.04, 0.09)
Lumori et al [10] 195 31 0.16 (0.11, 0.22)
Shogade et al [12] 134 8 0.06 (0.03, 0.11)
Tremamunno et al [13] 84 10 0.12 (0.06, 0.21)
Zhen et al [14] 108 10 0.09 (0.05, 0.16)
Random−effects 0.11 (0.07, 0.17)
Heterogeneity: I^2=89%, p<0.01
General population
Demmer et al [15] 511 228 0.45 (0.40, 0.49)
Population not specified
Kasha et al [18] 50 15 0.30 (0.18, 0.45)
Random−effects 0.15 (0.09, 0.25)
Heterogeneity: I^2=96%, p<0.01
Test for subgroup differences: x^2_2=51.68, df=2 (p<0.01)
0 0.1 0.2 0.3 0.4 0.5 0.6
Prevalence (%)

C.

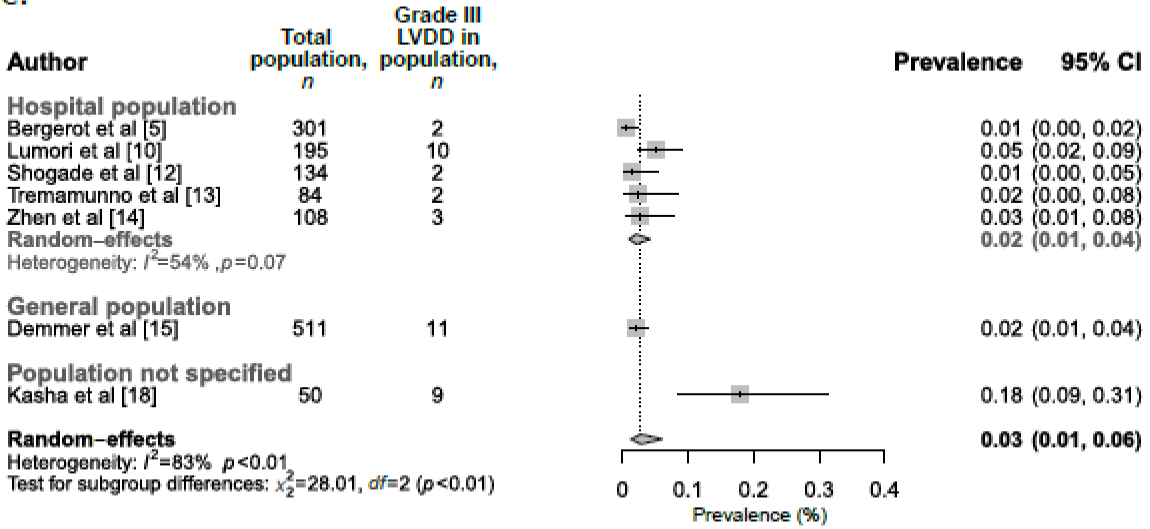

Author
Total population, n
Grade III LVDD in population, n
Prevalence
95% CI
Hospital population
Bergerot et al [5] 301 2 0.01 (0.00, 0.02)
Lumori et al [10] 195 10 0.05 (0.02, 0.09)
Shogade et al [12] 134 2 0.01 (0.00, 0.05)
Tremamunno et al [13] 84 2 0.02 (0.00, 0.08)
Zhen et al [14] 108 3 0.03 (0.01, 0.08)
Random−effects 0.02 (0.01, 0.04)
Heterogeneity: I^2=54% ,p=0.07
General population
Demmer et al [15] 511 11 0.02 (0.01, 0.04)
Population not specified
Kasha et al [18] 50 9 0.18 (0.09, 0.31)
Random−effects 0.03 (0.01, 0.06)
Heterogeneity: I^2=83% p<0.01
Test for subgroup differences: χ^2_2=28.01, df=2 (p<0.01)
0
0.1
0.2
0.3
0.4
Prevalence (%)

ESM Figure 3: Prevalence of LVDD in individuals with type 2 diabetes in the hospital and general population, categorised as (a) indeterminate or (b) definitive based on American society of Echocardiography/European Association of Cardiovascular Imaging (ASE/EACVI) recommendations, in studies using a LVEF cut-off of ≥50%. df, degrees of freedom.

a.

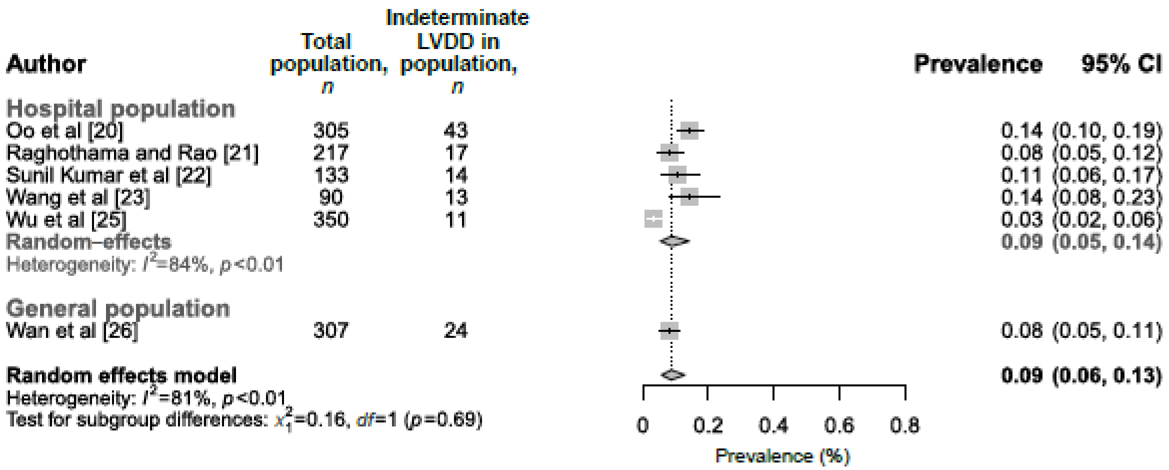

b.

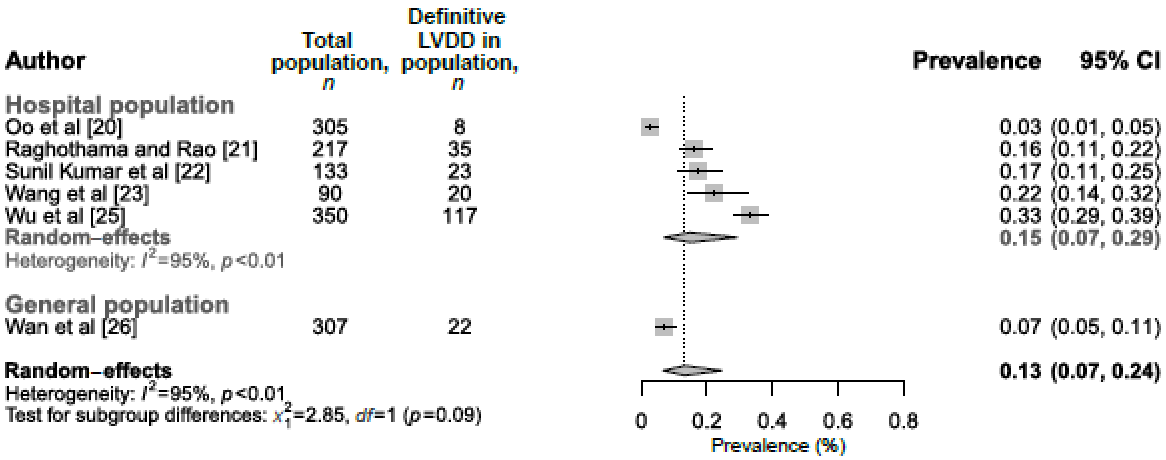
Author
Total population, n
Definitive LVDD in population, n
Prevalence
95% CI
Hospital population
Oo et al [20] 305 8 0.03 (0.01, 0.05)
Raghothama and Rao [21] 217 35 0.16 (0.11, 0.22)
Sunil Kumar et al [22] 133 23 0.17 (0.11, 0.25)
Wang et al [23] 90 20 0.22 (0.14, 0.32)
Wu et al [25] 350 117 0.33 (0.29, 0.39)
Random−effects 0.15 (0.07, 0.29)
Heterogeneity: I^2=95%, p<0.01
General population
Wan et al [26] 307 22 0.07 (0.05, 0.11)
Random−effects 0.13 (0.07, 0.24)
Heterogeneity: I^2=95%, p<0.01
Test for subgroup differences: χ_1^2=2.85, df=1 (p=0.09)
0 0.2 0.4 0.6 0.8
Prevalence (%)

References

1. Bouthoorn S, Gohar A, Valstar G, den Ruijter HM, Reitsma JB, Hoes AW, et al. Prevalence of left ventricular systolic dysfunction and heart failure with reduced ejection fraction in men and women with type 2 diabetes mellitus: a systematic review and meta-analysis. Cardiovasc Diabetol. 2018;17(1):58.
2. Bouthoorn S, Valstar GB, Gohar A, den Ruijter HM, Reitsma HB, Hoes AW, Rutten FH. The prevalence of left ventricular diastolic dysfunction and heart failure with preserved ejection fraction in men and women with type 2 diabetes: A systematic review and meta-analysis. Diab Vasc Dis Res. 2018;15(6):477-93.
3. Hoy D, Brooks P, Woolf A, Blyth F, March L, Bain C, et al. Assessing risk of bias in prevalence studies: modification of an existing tool and evidence of interrater agreement. J Clin Epidemiol. 2012;65(9):934-9.
4. Barendregt JJ, Doi SA, Lee YY, Norman RE, Vos T. Meta-analysis of prevalence. J Epidemiol Community Health. 2013;67(11):974-8.
5. Bergerot C, Davidsen ES, Amaz C, Thibault H, Altman M, Bellaton A, et al. Diastolic function deterioration in type 2 diabetes mellitus: predictive factors over a 3-year follow-up. Eur Heart J Cardiovasc Imaging. 2018;19(1):67-73.
6. Nagueh SF, Appleton CP, Gillebert TC, Marino PN, Oh JK, Smiseth OA, et al. Recommendations for the evaluation of left ventricular diastolic function by echocardiography. J Am Soc Echocardiogr. 2009;22(2):107-33.
7. Chee KH, Tan KL, Luqman I, Saiful SS, Chew YY, Chinna K, Tan ATB. Prevalence and Predictors of Left Ventricular Diastolic Dysfunction in Malaysian Patients With Type 2 Diabetes Mellitus Without Prior Known Cardiovascular Disease. Front Cardiovasc Med. 2021;8:676862.
8. Nagueh SF, Smiseth OA, Appleton CP, Byrd BF, 3rd, Dokainish H, Edvardsen T, et al. Recommendations for the Evaluation of Left Ventricular Diastolic Function by Echocardiography: An Update from the American Society of Echocardiography and the European Association of Cardiovascular Imaging. J Am Soc Echocardiogr. 2016;29(4):277-314.
9. Joseph TP, Kotecha NS, Kumar HBC, Jain N, Kapoor A, Kumar S, et al. Coronary artery calcification, carotid intima-media thickness and cardiac dysfunction in young adults with type 2 diabetes mellitus. J Diabetes Complications. 2020;34(8):107609.
10. Lumori BAE, Nuwagira E, Abeya FC, Araye AA, Masette G, Mondo CK, et al. Association of body mass index with left ventricular diastolic dysfunction among ambulatory individuals with diabetes mellitus in rural Uganda: a cross-sectional study. BMC Cardiovasc Disord. 2022;22(1):279.
11. Nasir MH, S.N.; Hassan, A. Frequency of left ventricle diastolic dysfunction in asymptomatic type II diabetic patients. Pakistan Journal of Medical and Health Sciences; 2016. p. 905-8.
12. Shogade TT, Essien IO, Ekrikpo UE, Umoh IO, Utin CT, Unadike BC, Andy JJ. Association of microalbuminuria with left ventricular dysfunction in Nigerian normotensive type 2 diabetes patients. Cardiovasc J Afr. 2018;29(5):283-8.
13. Tremamunno S, De Vita A, Villano A, Melita V, Ingrasciotta G, Ruscio E, et al. Relation of endothelial and cardiac autonomic function with left ventricle diastolic function in patients with type 2 diabetes mellitus. Diabetes Metab Res Rev. 2022;38(2):e3484.
14. Zhen Z, Chen Y, Liu JH, Chan CW, Yuen M, Lam KS, et al. Increased T-wave alternans is associated with subclinical myocardial structural and functional abnormalities in patients with type 2 diabetes. J Cardiol. 2016;68(4):329-34.
15. Demmer RT, Allison MA, Cai J, Kaplan RC, Desai AA, Hurwitz BE, et al. Association of Impaired Glucose Regulation and Insulin Resistance With Cardiac Structure and Function: Results From ECHO-SOL (Echocardiographic Study of Latinos). Circ Cardiovasc Imaging. 2016;9(10).

16. Ommen SR, Nishimura RA, Appleton CP, Miller FA, Oh JK, Redfield MM, Tajik AJ. Clinical utility of Doppler echocardiography and tissue Doppler imaging in the estimation of left ventricular filling pressures: A comparative simultaneous Doppler-catheterization study. Circulation. 2000;102(15):1788-94.
17. Klajda MD, Scott CG, Rodeheffer RJ, Chen HH. Diabetes Mellitus Is an Independent Predictor for the Development of Heart Failure: A Population Study. Mayo Clin Proc. 2020;95(1):124-33.
18. Kasha AM, B.; Noorul Ameen, K.H. Study to detect the prevalence of LV diastolic dysfunction in diabetic patients aged less than or equal to 50 years in a tertiary care center. Research Journal of Pharmaceutical, Biological and Chemical Sciences. 2017;8(4):26-32.
19. Yang H, Wang Y, Negishi K, Nolan M, Marwick TH. Pathophysiological effects of different risk factors for heart failure. Open Heart. 2016;3(1):e000339.
20. Oo MM, Tan Chung Zhen I, Ng KS, Tan KL, Tan ATB, Vethakkan SR, et al. Observational study investigating the prevalence of asymptomatic stage B heart failure in patients with type 2 diabetes who are not known to have coronary artery disease. BMJ Open. 2021;11(1):e039869.
21. Raghothama SR, A. Revelation of subclinical left ventricular diastolic dysfunction in patients with type 2 diabetes mellitus using 2016 ASE/ EACVI guidelines. Caspian Journal of Internal Medicine. 2021;12:586-92.
22. Sunil Kumar SD, N.; Gona, O.J.; Vinay Kumar, K.; Madhu, B. Impact of updated 2016 ASE/EACVI VISÀ-VIS 2009 ASE recommendation on the prevalence of diastolic dysfunction and Lv filling pressures in patients with preserved ejection fraction. Journal of Cardiovascular Imaging. 2021;29:31-43.
23. Wang YC, S.; You, C. APPLICATION VALUE OF FOUR-DIMENSIONAL LEFT ATRIAL STRAIN IN EVALUATING THE LEFT VENTRICULAR DIASTOLIC FUNCTION OF T2DM. Acta Medica Mediterranea. 2022;38:979-84.
24. Yang SY, Hwang HJ. Does diabetes increase the risk of cardiovascular events in patients with negative treadmill stress echocardiography? Endocr J. 2022;69(7):785-96.
25. Wu MZ, Chen Y, Yu YJ, Zhen Z, Liu YX, Zou Y, et al. Sex-specific pattern of left ventricular hypertrophy and diastolic function in patients with type 2 diabetes mellitus. Eur Heart J Cardiovasc Imaging. 2021;22(8):930-40.
26. Wan SH, Pumerantz AS, Dong F, Ochoa C, Chen HH. Comparing the influence of 2009 versus 2016 ASE/EACVI diastolic function guidelines on the prevalence and echocardiographic characteristics of preclinical diastolic dysfunction (stage B heart failure) in a Hispanic population with type 2 diabetes mellitus. J Diabetes Complications. 2019;33(8):579-84.
27. Alhibaly HAA-J, H.H.; Bdair, B.W.H.; Algraittee, S.J.R. Evaluation of clinically significant cardiac abnormalities in patients with normal electrocardiogram using transthoracic echocardiography. Acta Medica Iranica. 2021;59(97-107).
28. Antakly-Hanon Y, Ben Hamou A, Garçon P, Moeuf Y, Banu I, Fumery M, et al. Asymptomatic left ventricular dysfunction in patients with type 2 diabetes free of cardiovascular disease and its relationship with clinical characteristics: The DIACAR cohort study. Diabetes Obes Metab. 2021;23(2):434-43.
29. Bayat FK, M.; Saffarian, F.; Shahrbaf, M.A. Correlation between type ii diabetes mellitus and left atrial function as assessed by 2d speckle-tracking echocardiography in patients without coronary artery disease. Iranian Heart Journal 2020;21(1):82-93.
30. Cioffi G, Giorda CB, Lucci D, Nada E, Ognibeni F, Mancusi C, et al. Effects of linagliptin on left ventricular DYsfunction in patients with type 2 DiAbetes and concentric left ventricular geometry: results of the DYDA 2 trial. Eur J Prev Cardiol. 2021;28(1):8-17.
31. Huang Z, Zhong J, Zhang S, Xiong Z, Huang Y, Liu M, et al. Association between serum cystatin C and early impairment of cardiac function and structure in type 2 diabetes patients with normal renal function. Clin Cardiol. 2022.

32. Ibrahim IMA-K, A.-R.O.; Mahfouz, R.A.; Shehata, I.E. The Association Between Morning Blood Pressure Surge and Cardiovascular Disease in Normotensive Type 2 Diabetic Patients: Observational Analytical Study in the Form of a Cross-Sectional Study. SN Comprehensive Clinical Medicine. 2021;3(4):955-63.
33. Liu YM, J.; Guo, J.; Lu, H.; Zhang, Y.; Chen, Y. Characteristics of myocardial perfusion in type 2 diabetes mellitus and its association with left ventricular diastolic dysfunction: A study of myocardial contrast echocardiography. International Journal of General Medicine. 2021(14):7533-43.
34. Lu Z, Lense L, Sharma M, Shah A, Luu Y, Cardinal L, et al. Prevalence of QT prolongation and associated LVEF changes in diabetic patients over a four-year retrospective time period. J Community Hosp Intern Med Perspect. 2017;7(2):87-94.
35. Maiello M, Zito A, Carbonara S, Ciccone MM, Palmiero P. Left ventricular mass, geometry and function in diabetic patients affected by coronary artery disease. J Diabetes Complications. 2017;31(10):1533-7.
36. Patro PKD, B.K.; Choudhury, S.; Sethy, R.C. Study of microalbuminuria in type 2 diabetes mellitus as a predictor of left ventricular dysfunction- a cohort study. Journal of Clinical and Diagnostic Research. 2021;15:OC05-OC9.
37. Qureshi SAP, S.A.R.; Naeem, H.A.G.; Qazi, M.A. Frequency of left ventricular diastolic dysfunction among type-II diabetics with non-alcoholic fatty liver disease. Pakistan Journal of Medical and Health Sciences; 2016. p. 200-2.
38. Shahapure PS, S. Cardiovascular morbidity in diabetic non-alcoholic fatty liver disease (Nafld) using nafld fibrosis score as an early indicator. Turkish Journal of Endocrinology and Metabolism. 2020;24:314-20.
39. Wang ZW, C.; Xie, Z.; Huang, X.; ShangGuan, H.; Zhu, W.; Wang, S. Echocardiographic phenotypes of Chinese patients with type 2 diabetes may indicate early diabetic myocardial disease. ESC Heart Failure. 2022.
40. Zoppini GB, C.; Bonapace, S.; Trombetta, M.; Mantovani, A.; Toffalini, A.; Lanzoni, L.; Bertolini, L.; Zenari, L.; Bonora, E.; Targher, G.; Rossi, A. Left ventricular chamber dilation and filling pressure may help to categorise patients with type 2 diabetes. BMJ Open Diabetes Research and Care. 2018;6.
41. Zuo X, Liu X, Chen R, Ou H, Lai J, Zhang Y, Yan D. An in-depth analysis of glycosylated haemoglobin level, body mass index and left ventricular diastolic dysfunction in patients with type 2 diabetes. BMC Endocr Disord. 2019;19(1):88.
42. Alizadehasl AB, H.; Sadeghipour, P.; Mohebbi, B.; Baay, M.; Alemzadeh-Ansari, M.J.; Hosseini, Z.; Norouzi, Z.; Maleki, M.; Noohi, F.; Khalili, Y.; Naderi, N.; Pouraliakbar, H.; Ghadrdoost, B.; Arabian, M.; Khaleghparast, S.; Boudagh, S. Normal values of echocardiographic parameters and incidence of valvular heart findings in 2229 iranian peaple: Results from the 'HAMRAH survey'. Research in Cardiovascular Medicine. 2021;10(2):45-53.
43. Kim SH, Sung KC, Lee SK, Park J, Kim NH, Kim SH, Shin C. Longitudinal changes in left ventricular structure and function in patients with type 2 diabetes: Normal weight versus overweight/obesity. Diab Vasc Dis Res. 2019;16(5):450-7.
44. Lee H, Kim G, Choi YJ, Huh BW, Lee BW, Kang ES, et al. Association between Non-Alcoholic Steatohepatitis and Left Ventricular Diastolic Dysfunction in Type 2 Diabetes Mellitus. Diabetes Metab J. 2020;44(2):267-76.
45. Lee KA, Kim YJ, Park TS, Lee JH, Jin HY. The association between cardiac autonomic neuropathy and heart function in type 2 diabetic patients. Somatosens Mot Res. 2020;37(3):149-56.
46. Segar MW, Khan MS, Patel KV, Butler J, Tang WHW, Vaduganathan M, et al. Prevalence and Prognostic Implications of Diabetes With Cardiomyopathy in Community-Dwelling Adults. J Am Coll Cardiol. 2021;78(16):1587-98.

47. Wang Y, Yang H, Huynh Q, Nolan M, Negishi K, Marwick TH. Diagnosis of Nonischemic Stage B Heart Failure in Type 2 Diabetes Mellitus: Optimal Parameters for Prediction of Heart Failure. JACC Cardiovasc Imaging. 2018;11(10):1390-400.
48. Shaker A. Exercise electrocardiogram testing in asymptomatic patient with type 2 diabetes and left ventricular diastolic dysfunction. Journal of Indian College of Cardiology. 2019;9:205-10.
49. Gimeno-Orna JAR-P, L.; Anguita-Sánchez, M.; Barrios, V.; Muñiz, J.; Pérez, A. Association of the kdigo risk classification with the prevalence of heart failure in patients with type 2 diabetes. Journal of Clinical Medicine. 2021;10.
50. Ponikowski P, Voors AA, Anker SD, Bueno H, Cleland JG, Coats AJ, et al. 2016 ESC Guidelines for the diagnosis and treatment of acute and chronic heart failure: The Task Force for the diagnosis and treatment of acute and chronic heart failure of the European Society of Cardiology (ESC). Developed with the special contribution of the Heart Failure Association (HFA) of the ESC. Eur J Heart Fail. 2016;18(8):891-975.
51. Zhou M, Huang D, Cheng Y, Lau YM, Lai WH, Lau YM, et al. Opportunistic screening for asymptomatic left ventricular dysfunction in type 2 diabetes mellitus. Postgrad Med J. 2022.
52. Ianoș RD, Pop C, Iancu M, Rahaian R, Cozma A, Procopciuc LM. Diagnostic Performance of Serum Biomarkers Fibroblast Growth Factor 21, Galectin-3 and Copeptin for Heart Failure with Preserved Ejection Fraction in a Sample of Patients with Type 2 Diabetes Mellitus. Diagnostics (Basel). 2021;11(9).
53. Pieske B, Tschope C, de Boer RA, Fraser AG, Anker SD, Donal E, et al. How to diagnose heart failure with preserved ejection fraction: the HFA-PEFF diagnostic algorithm: a consensus recommendation from the Heart Failure Association (HFA) of the European Society of Cardiology (ESC). Eur Heart J. 2019;40(40):3297-317.
54. Jensen J, Schou M, Kistorp C, Faber J, Hansen TW, Jensen MT, et al. Prevalence of heart failure and the diagnostic value of MR-proANP in outpatients with type 2 diabetes. Diabetes Obes Metab. 2019;21(3):736-40.
55. Li J, Wu N, Dai W, Jiang L, Li Y, Li S, Wen Z. Association of serum calcium and heart failure with preserved ejection fraction in patients with type 2 diabetes. Cardiovasc Diabetol. 2016;15(1):140.
