# Supplementary material for: Epidemiology of heart failure in diabetes: a disease in disguise
Source: Diabetologia. 2024 Feb 9;67(4):574–601. doi: 10.1007/s00125-023-06068-2 (PMC10904471; doi:10.1007/s00125-023-06068-2)
Supplement: Supplementary file 2 — Supplementary file2 (PPTX 1.43 MB) [file 125_2023_6068_MOESM2_ESM.pptx]

## Slide 1
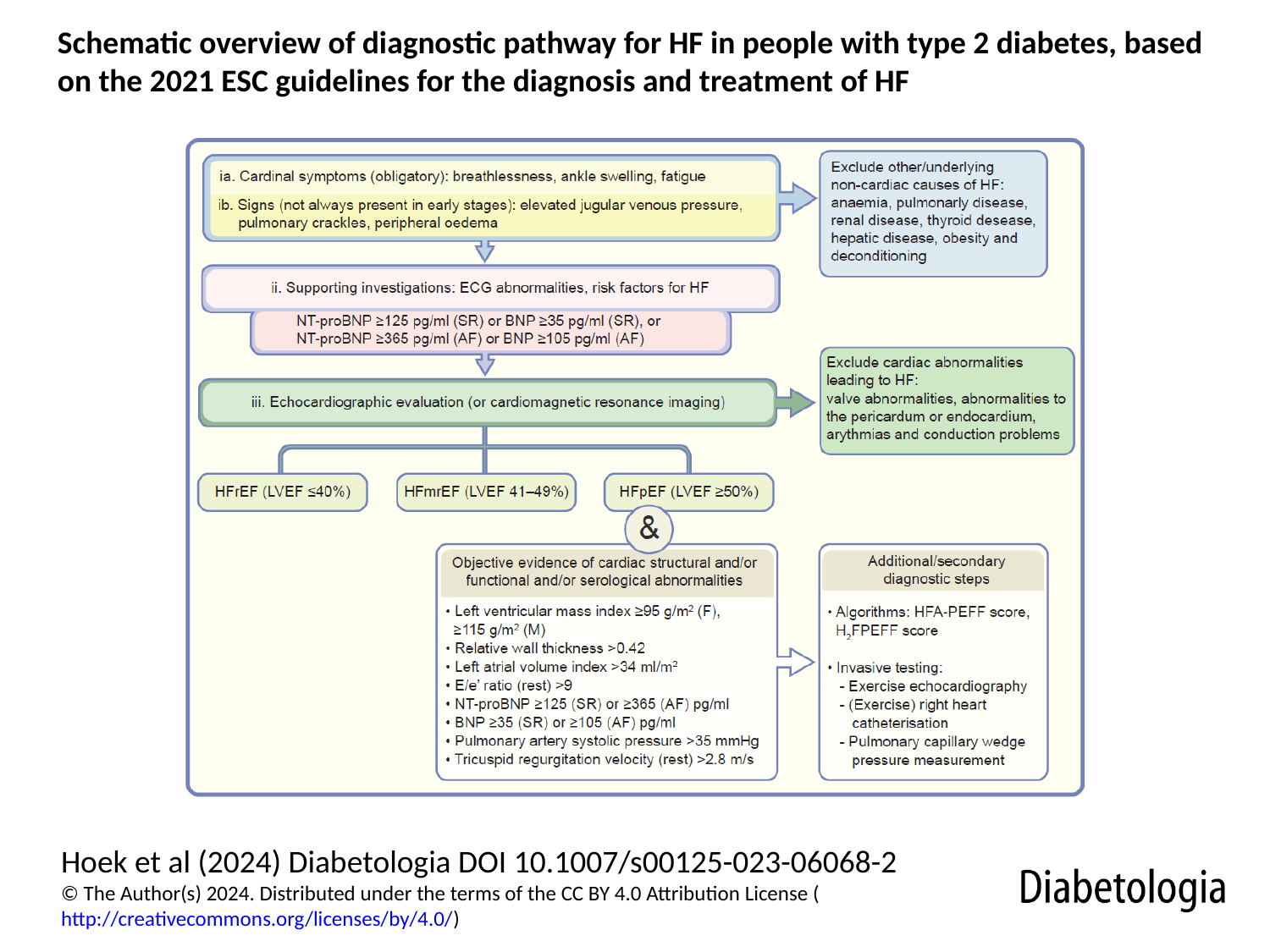

Schematic overview of diagnostic pathway for HF in people with type 2 diabetes, based on the 2021 ESC guidelines for the diagnosis and treatment of HF
Hoek et al (2024) Diabetologia DOI 10.1007/s00125-023-06068-2
© The Author(s) 2024. Distributed under the terms of the CC BY 4.0 Attribution License (http://creativecommons.org/licenses/by/4.0/)

## Slide 2
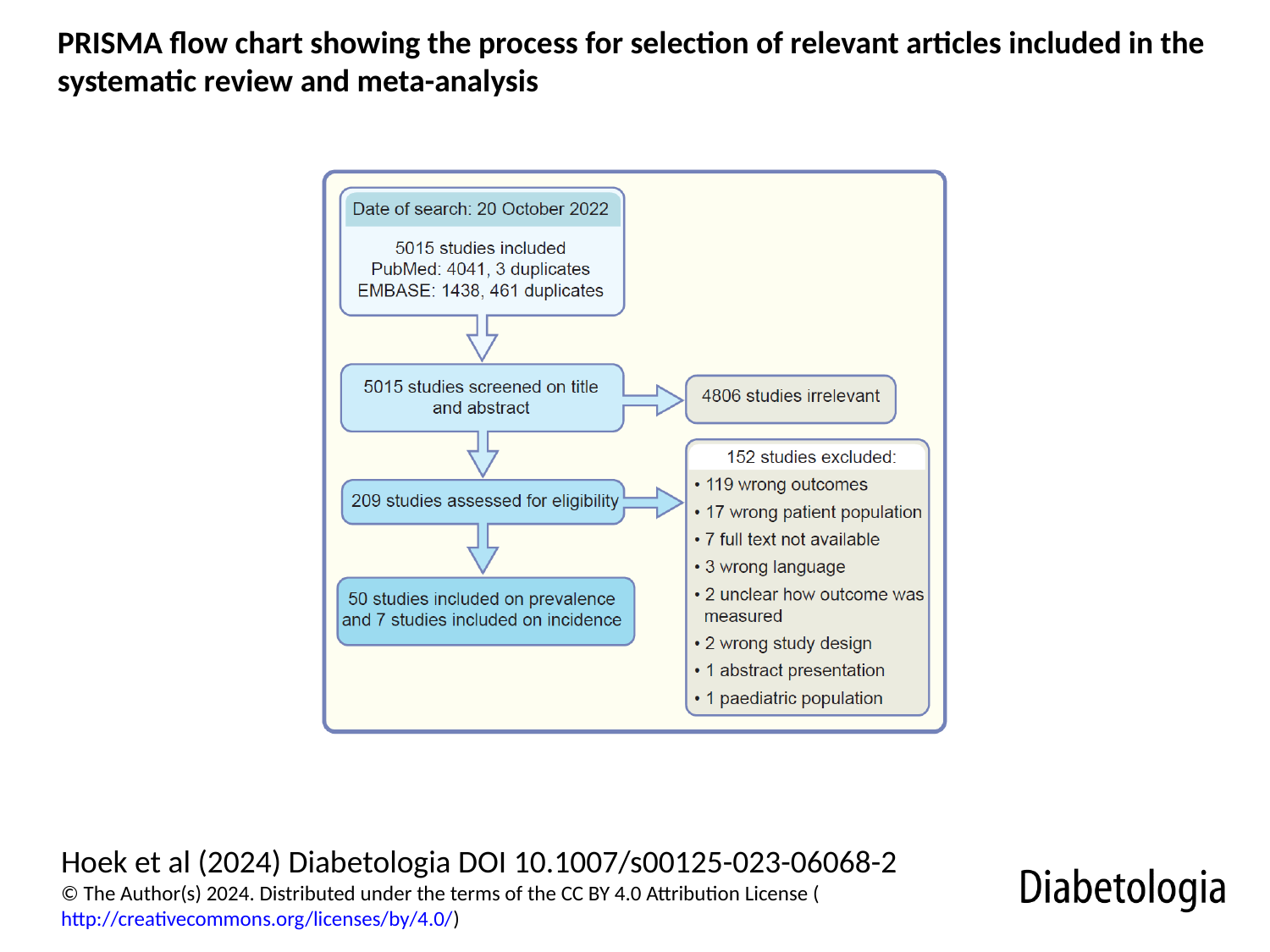

PRISMA flow chart showing the process for selection of relevant articles included in the systematic review and meta-analysis
Hoek et al (2024) Diabetologia DOI 10.1007/s00125-023-06068-2
© The Author(s) 2024. Distributed under the terms of the CC BY 4.0 Attribution License (http://creativecommons.org/licenses/by/4.0/)

## Slide 3
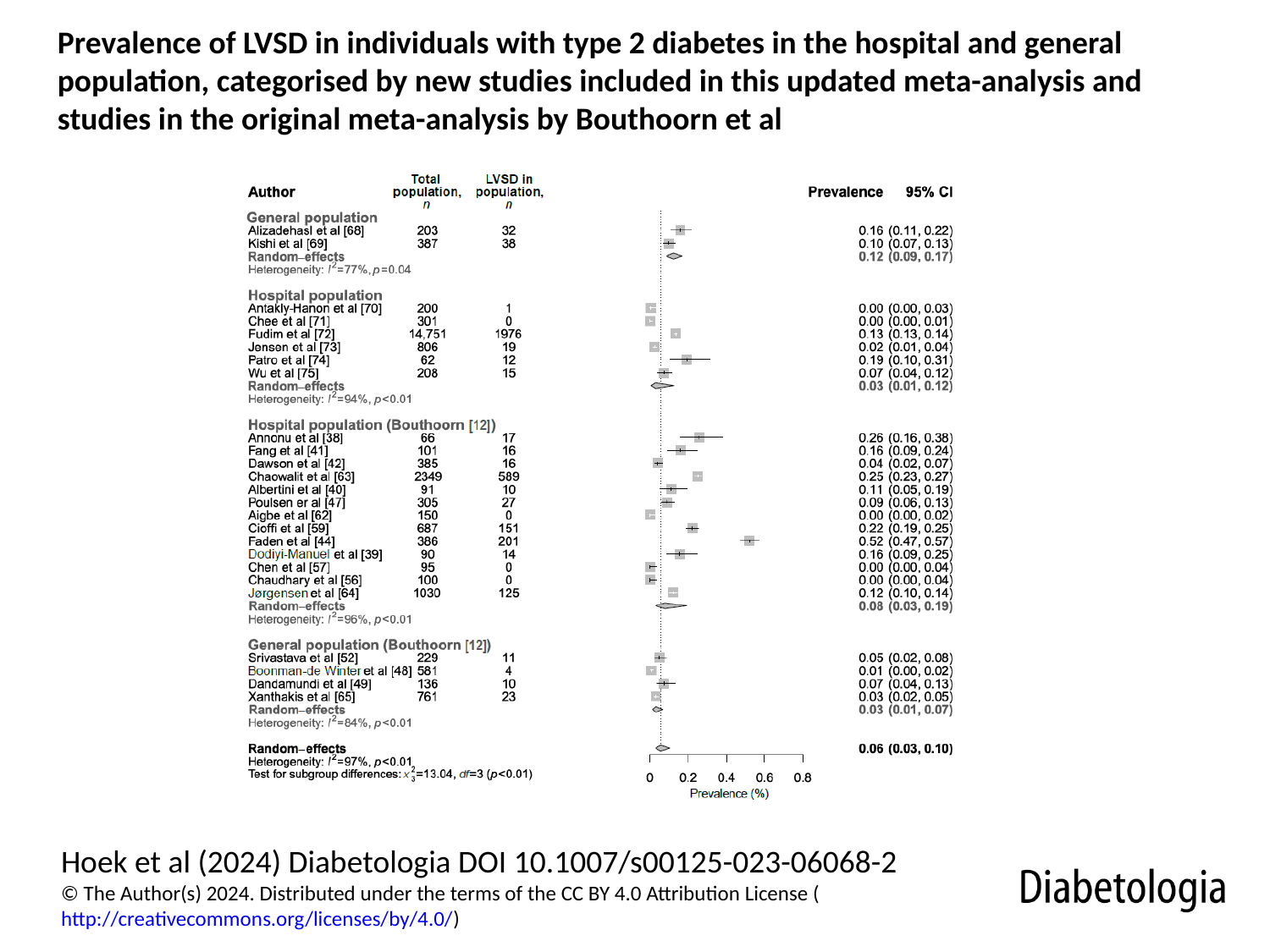

Prevalence of LVSD in individuals with type 2 diabetes in the hospital and general population, categorised by new studies included in this updated meta-analysis and studies in the original meta-analysis by Bouthoorn et al
Hoek et al (2024) Diabetologia DOI 10.1007/s00125-023-06068-2
© The Author(s) 2024. Distributed under the terms of the CC BY 4.0 Attribution License (http://creativecommons.org/licenses/by/4.0/)

## Slide 4
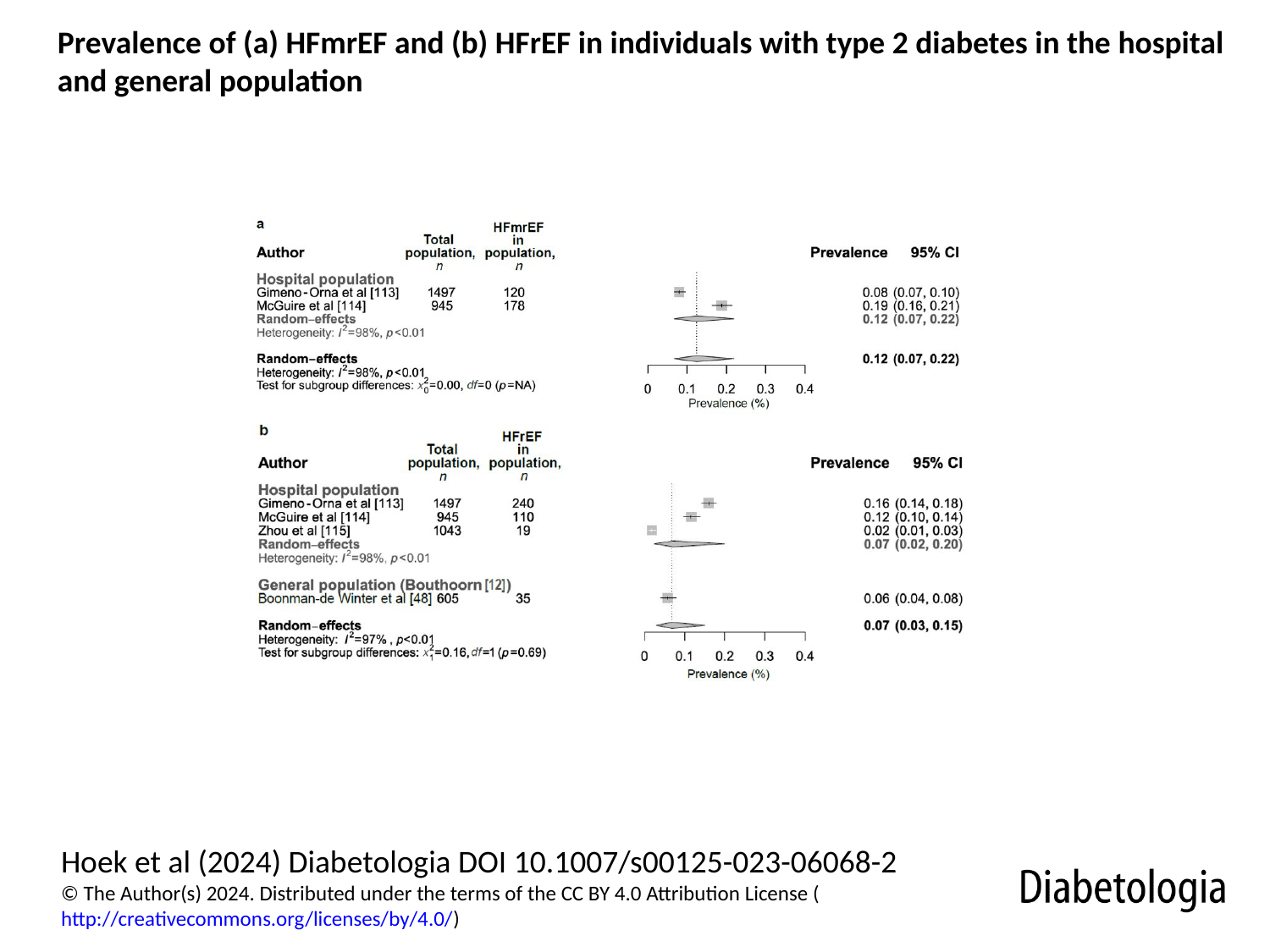

Prevalence of (a) HFmrEF and (b) HFrEF in individuals with type 2 diabetes in the hospital and general population
Hoek et al (2024) Diabetologia DOI 10.1007/s00125-023-06068-2
© The Author(s) 2024. Distributed under the terms of the CC BY 4.0 Attribution License (http://creativecommons.org/licenses/by/4.0/)

## Slide 5
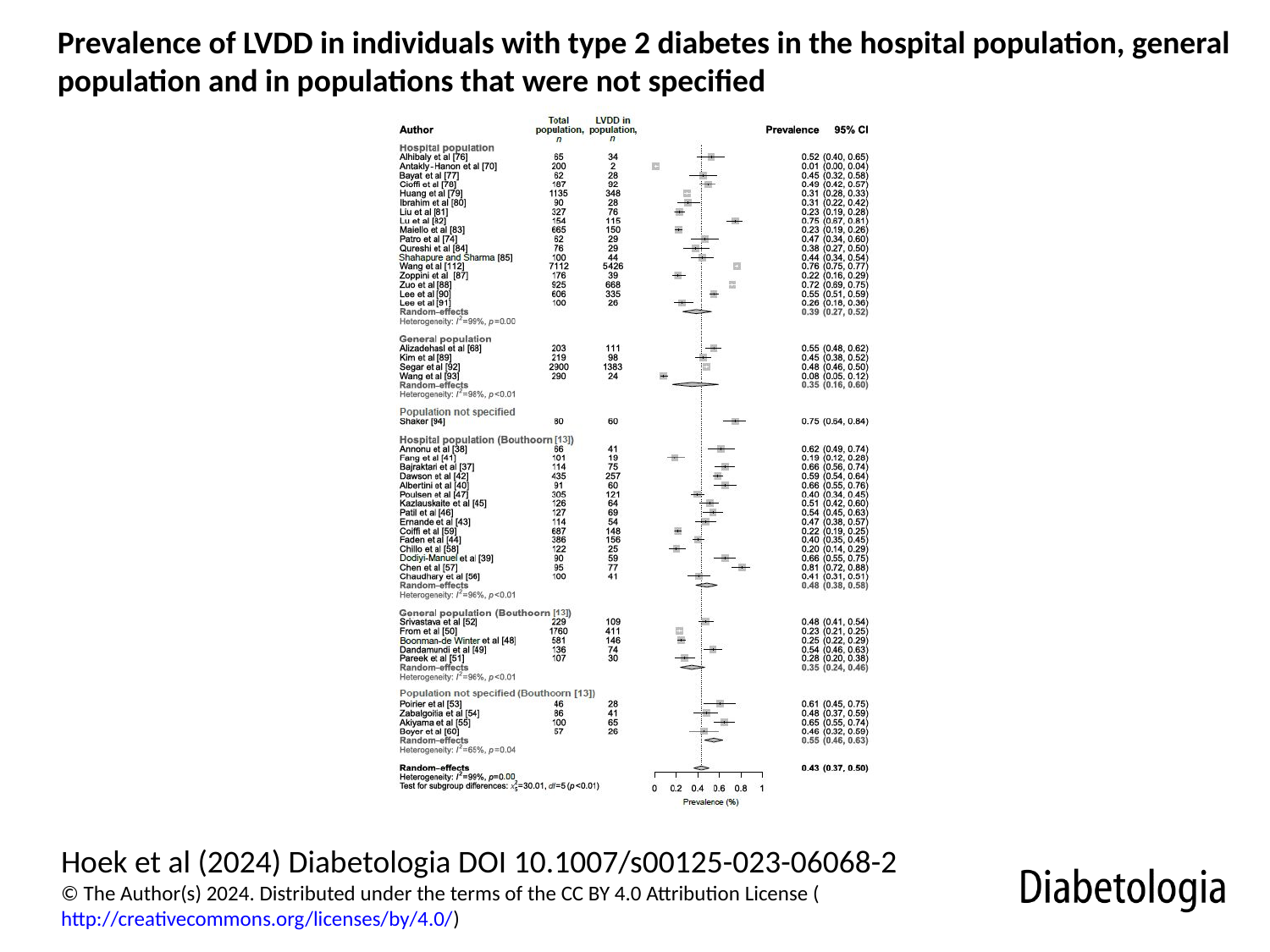

Prevalence of LVDD in individuals with type 2 diabetes in the hospital population, general population and in populations that were not specified
Hoek et al (2024) Diabetologia DOI 10.1007/s00125-023-06068-2
© The Author(s) 2024. Distributed under the terms of the CC BY 4.0 Attribution License (http://creativecommons.org/licenses/by/4.0/)

## Slide 6
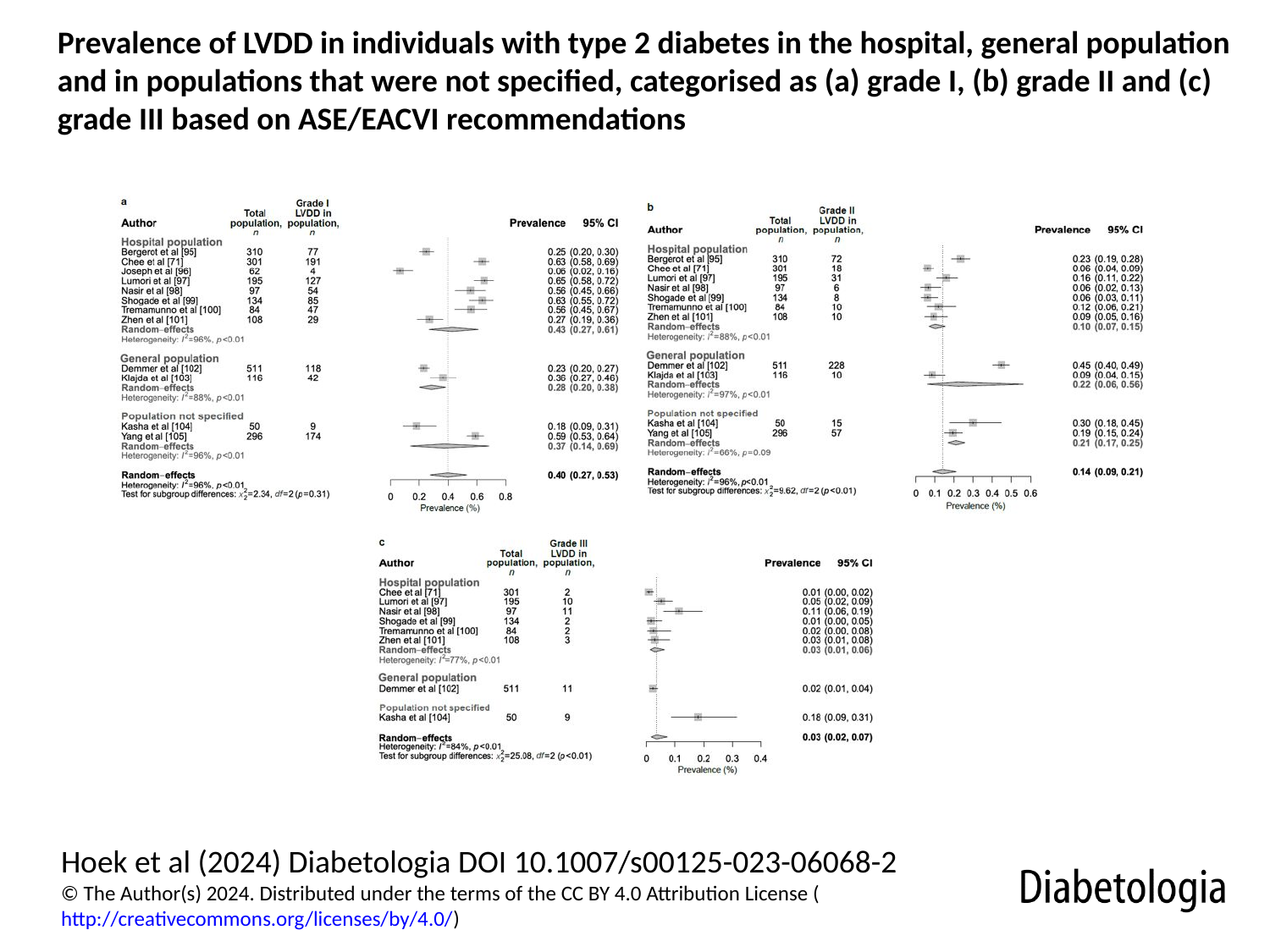

Prevalence of LVDD in individuals with type 2 diabetes in the hospital, general population and in populations that were not specified, categorised as (a) grade I, (b) grade II and (c) grade III based on ASE/EACVI recommendations
Hoek et al (2024) Diabetologia DOI 10.1007/s00125-023-06068-2
© The Author(s) 2024. Distributed under the terms of the CC BY 4.0 Attribution License (http://creativecommons.org/licenses/by/4.0/)

## Slide 7
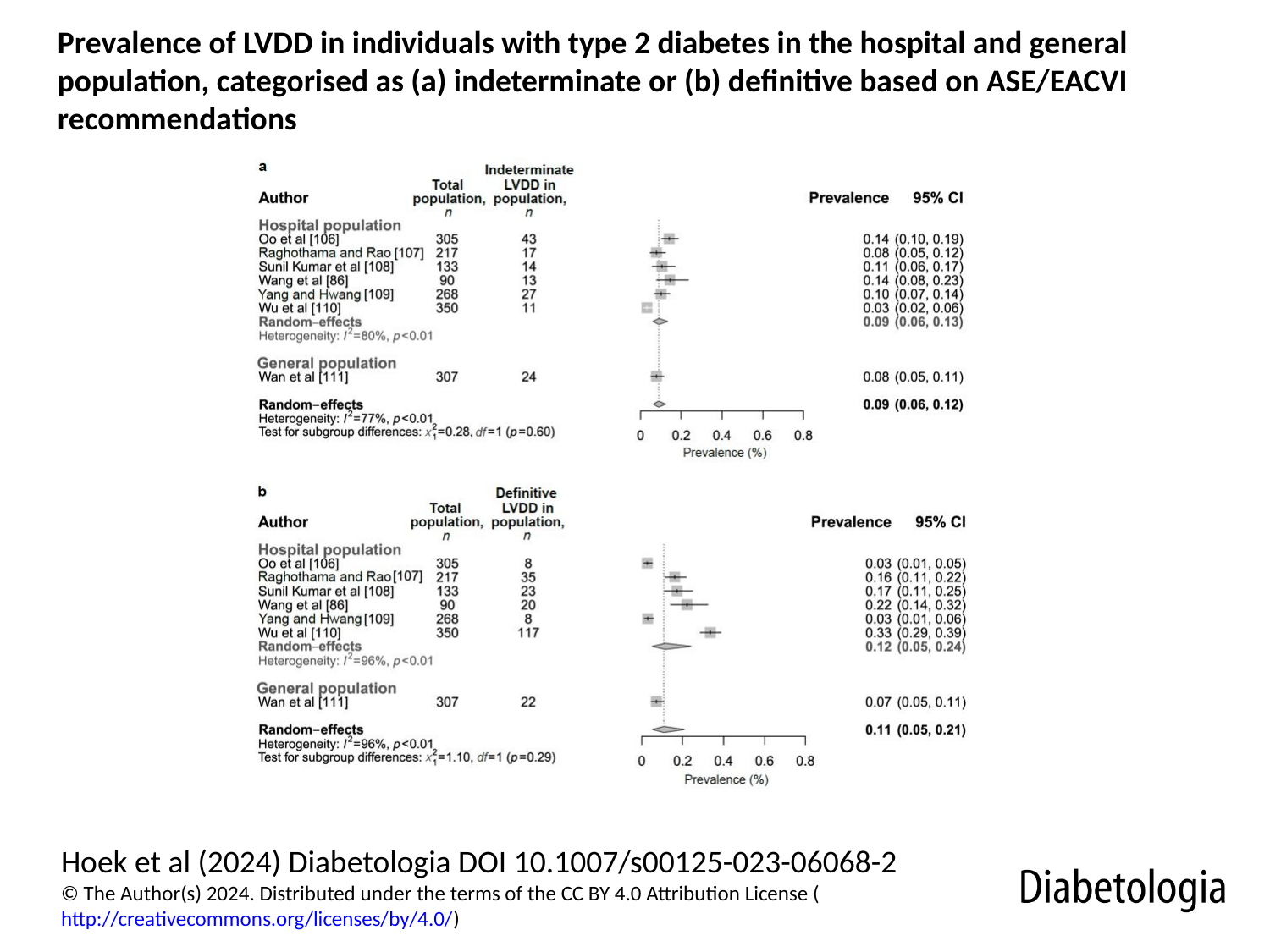

Prevalence of LVDD in individuals with type 2 diabetes in the hospital and general population, categorised as (a) indeterminate or (b) definitive based on ASE/EACVI recommendations
Hoek et al (2024) Diabetologia DOI 10.1007/s00125-023-06068-2
© The Author(s) 2024. Distributed under the terms of the CC BY 4.0 Attribution License (http://creativecommons.org/licenses/by/4.0/)

## Slide 8
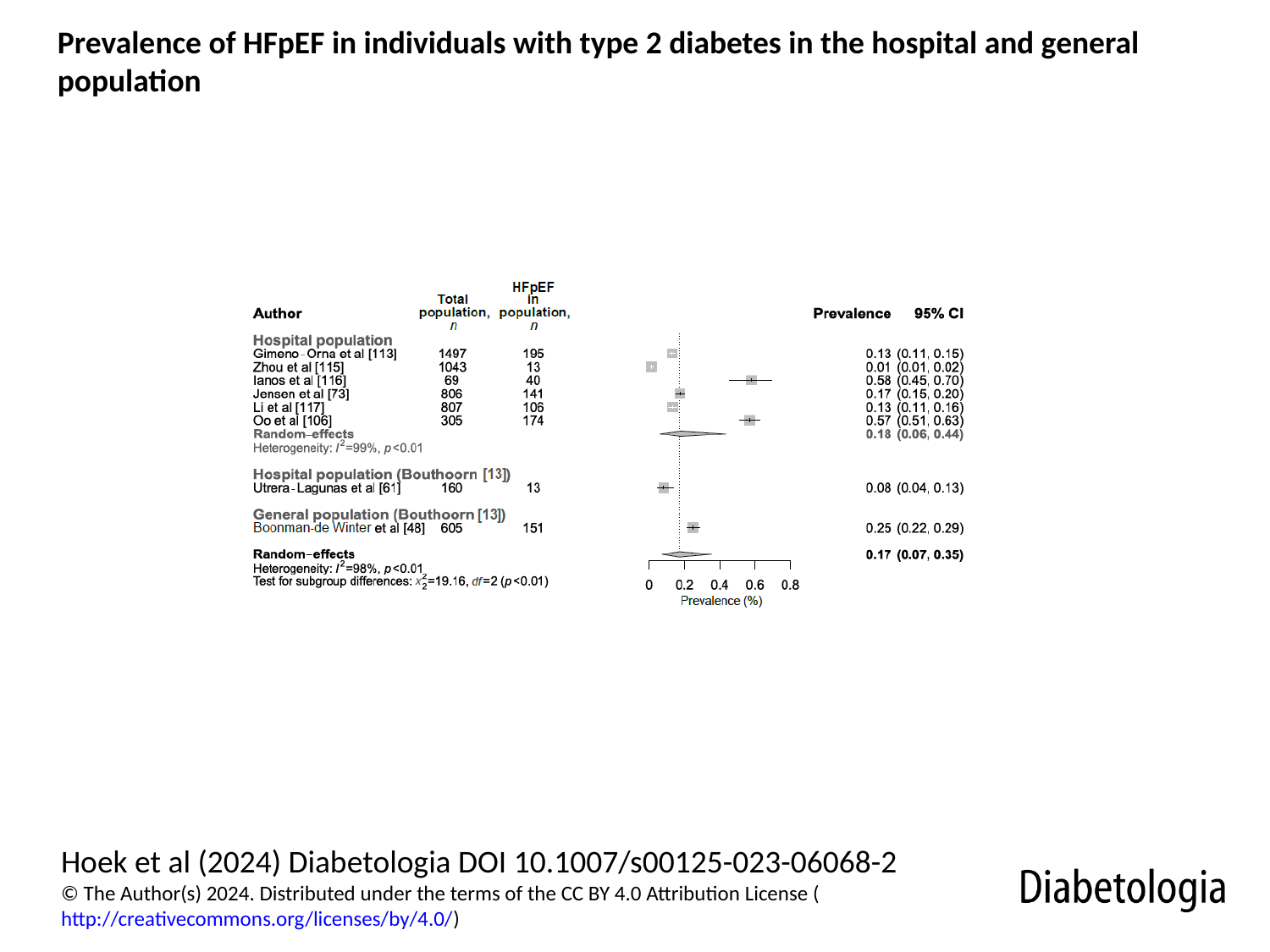

Prevalence of HFpEF in individuals with type 2 diabetes in the hospital and general population
Hoek et al (2024) Diabetologia DOI 10.1007/s00125-023-06068-2
© The Author(s) 2024. Distributed under the terms of the CC BY 4.0 Attribution License (http://creativecommons.org/licenses/by/4.0/)

## Slide 9
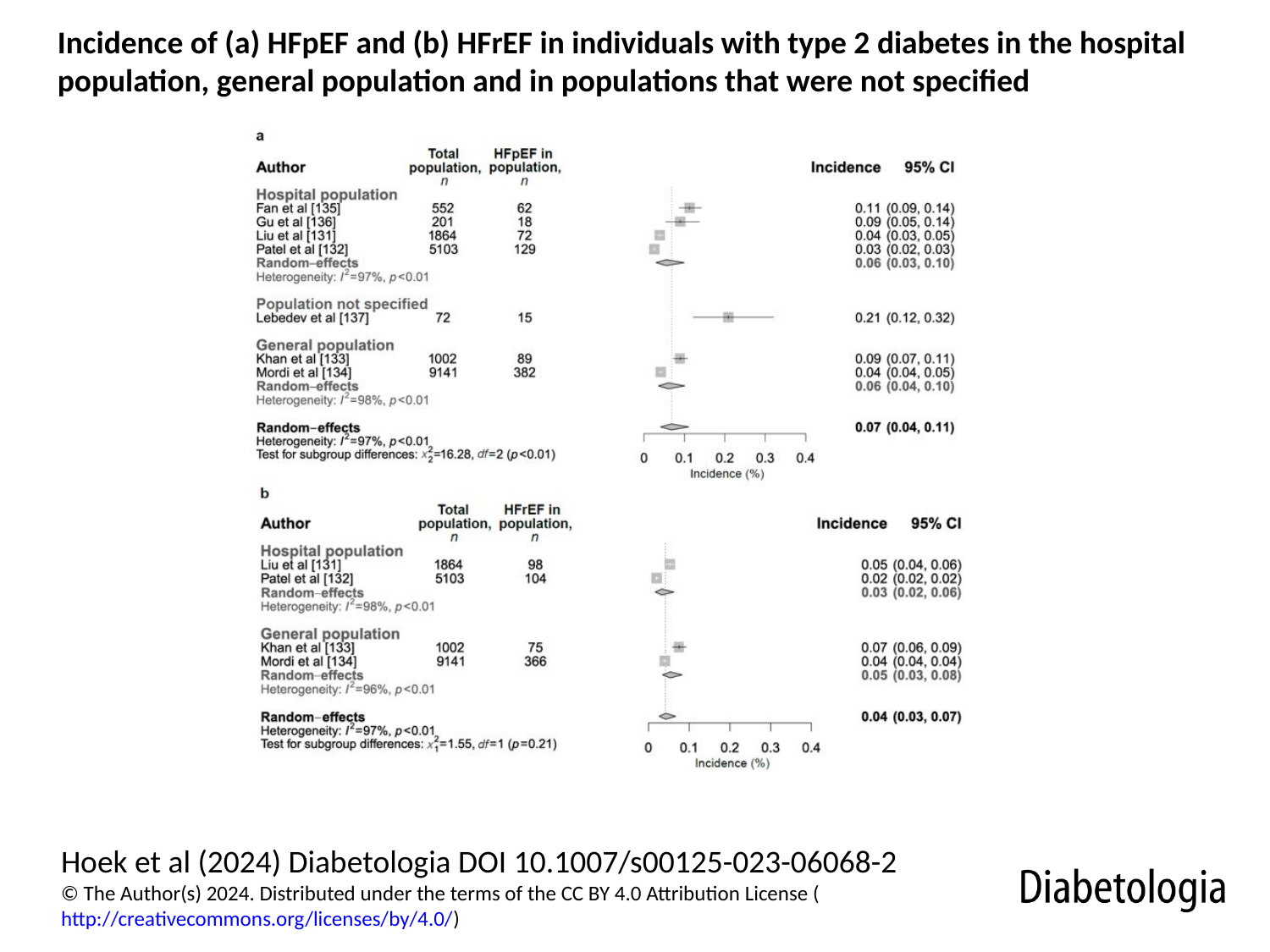

Incidence of (a) HFpEF and (b) HFrEF in individuals with type 2 diabetes in the hospital population, general population and in populations that were not specified
Hoek et al (2024) Diabetologia DOI 10.1007/s00125-023-06068-2
© The Author(s) 2024. Distributed under the terms of the CC BY 4.0 Attribution License (http://creativecommons.org/licenses/by/4.0/)
